# SGLT1 and SGLT2 inhibition, circulating metabolites, and cerebral small vessel disease: a mediation Mendelian Randomization study

**DOI:** 10.1186/s12933-024-02255-6

**Published:** 2024-05-07

**Authors:** Yanchen Lv, Xin Cheng, Qiang Dong

**Affiliations:** 1 Department of Neurology, National Center for Neurological Disorders, National Clinical Research Centre for Aging and Medicine, Huashan Hospital, Fudan University, Shanghai, China; 2 12 Wulumuqi Zhong Road, 200040 Shanghai, P. R. China

**Keywords:** Sodium-glucose cotransporter 2 inhibition, Sodium-glucose cotransporter 1 inhibition, Cerebral small vessel disease, Circulating metabolites, Mendelian Randomization

## Abstract

**Background:**

Sodium-glucose cotransporter 2 (SGLT2) and SGLT1 inhibitors may have additional beneficial metabolic effects on circulating metabolites beyond glucose regulation, which could contribute to a reduction in the burden of cerebral small vessel disease (CSVD). Accordingly, we used Mendelian Randomization (MR) to examine the role of circulating metabolites in mediating SGLT2 and SGLT1 inhibition in CSVD.

**Methods:**

Genetic instruments for SGLT1/2 inhibition were identified as genetic variants, which were both associated with the expression of encoding genes of SGLT1/2 inhibitors and glycated hemoglobin A1c (HbA1c) level. A two-sample two-step MR was used to determine the causal effects of SGLT1/2 inhibition on CSVD manifestations and the mediating effects of 1400 circulating metabolites linking SGLT1/2 inhibition with CSVD manifestations.

**Results:**

A lower risk of deep cerebral microbleeds (CMBs) and small vessel stroke (SVS) was linked to genetically predicted SGLT2 inhibition. Better white matter structure integrity was also achieved, as evidenced by decreased mean diffusivity (MD), axial diffusivity (AD), and radial diffusivity (RD), as well as lower deep (DWMH) and periventrivular white matter hyperintensity (PWMH) volume. Inhibiting SGLT2 could also lessen the incidence of severe enlarged perivascular spaces (EPVS) located at white matter, basal ganglia (BG) and hippocampus (HIP). SGLT1 inhibition could preserve white matter integrity, shown as decreased MD of white matter and DWMH volume. The effect of SGLT2 inhibition on SVS and MD of white matter through the concentration of 4-acetamidobutanoate and the cholesterol to oleoyl-linoleoyl-glycerol (18:1 to 18:2) ratio, with a mediated proportion of 30.3% and 35.5% of the total effect, respectively.

**Conclusions:**

SGLT2 and SGLT1 inhibition play protective roles in CSVD development. The SGLT2 inhibition could lower the risk of SVS and improve the integrity of white matter microstructure via modulating the level of 4-acetamidobutanoate and cholesterol metabolism. Further mechanistic and clinical studies research are needed to validate our findings.

**Supplementary Information:**

The online version contains supplementary material available at 10.1186/s12933-024-02255-6.

## Background

Cerebral small vessel disease (CSVD) accounts for nearly 20% of all ischemic strokes and is associated with a high risk of vascular dementia [1, 2]. Type-2 diabetes mellitus (T2DM) is a well-established risk factor for cerebrovascular diseases, such as ischemic stroke [3, 4]. Recent studies have suggested that T2DM is one of the major risk factor for microangiopathy and that patients with diabetes mellitus are more likely to suffer from CSVD [5, 6]. A comprehensive review revealed a substantial correlation between an elevated risk of small subcortical infarcts or white matter hyperintensity (WMH) and prediabetes and increased glycated hemoglobin A1c (HbA1c) level [7]. The Mendelian analysis and longitudinal research pointed out the causal impact of T2DM on CSVD progression [8–11]. Thus, it is crucial to alleviate CSVD in T2DM patients. Since the majority of diabetic patients take drugs to regulate their blood glucose levels, it is important to investigate whether long-term use of anti-diabetic medicines can prevent CSVD.

Sodium-glucose cotransporter 2 (SGLT2) inhibitors are a class of oral anti-diabetic drugs. They can lower serum glucose concentration by inhibiting glucose reabsorption in proximal tubules and promoting urinary glucose excretion [12]. Compelling evidence has shown their beneficial effects beyond glycemic control, but also in improving the prognosis of cardiovascular diseases, heart failure, and kidney diseases [13–16]. In addition, emerging reports have suggested that SGLT2 inhibitors have a neuroprotective effect on critical pathological alterations caused by CSVD, such as the loss of myelin and endothelial integrity [17]. According to recent research, the combination of SGLT1 and SGLT2 inhibitors may significantly reduce stroke risk [18]. The SGLT1 receptor can contribute to neuronal death during ischemia and eliminating it can prevent vascular dementia in a mouse model of CSVD [18, 19]. The above studies indicates that SGLT1/2 inhibitors can prevent CSVD, but the results are controversial, and the underlying metabolic mechanism remains unclear.

Metabolomics is an emerging systems biology technology that can be utilized to explore new biomarkers for disease detection and identify metabolic pathways associated with disease etiology [20]. Prior research has indicated that SGLT2 inhibitors can significantly affect the level of circulating metabolites, especially those related to lipids, amino acids, and ketone bodies [21–23]. Besides, mutations resulting in SGLT1 dysfunction may modify intestinal homeostasis and promote favorable metabolic outcomes [24, 25]. Increasing research has revealed a link between metabolic alternations and CSVD. The large-scale metabolomics study and Mendelian analysis found multiple metabolites associated with imaging markers of CSVD, cognition, and conversion to dementia [26–28]. Accordingly, there may exist metabolic pathways that mediate the impact of SGLT1/2 inhibitors on CSVD progression. However, there is a lack of large-scale longitudinal studies that can validate the causal relationship between the use of SGLT1/2 inhibitors and CSVD manifestations. Moreover, the role of circulating metabolites in the above association can only be partially verified due to the complexity of both the metabolomics and CSVD.

Mendelian Randomization (MR) can use related genetic variants as instrumental variables to examine causal associations, and it has been widely used to unveil the pathophysiological process of diseases in recent years [29]. In the present study, we first performed a two-sample MR study to examine the association between genetic proxies for SGLT2 and SGLT1 inhibitors and CSVD markers. Next, we applied a two-step MR analysis to establish the possible metabolic pathway from SGLT2 inhibition to CSVD using data on circulating metabolites. Our goal was to elucidate the metabolic changes that linked SGLT1/2 inhibition to CSVD.

## Methods

### Study design

**Fig. 1:**
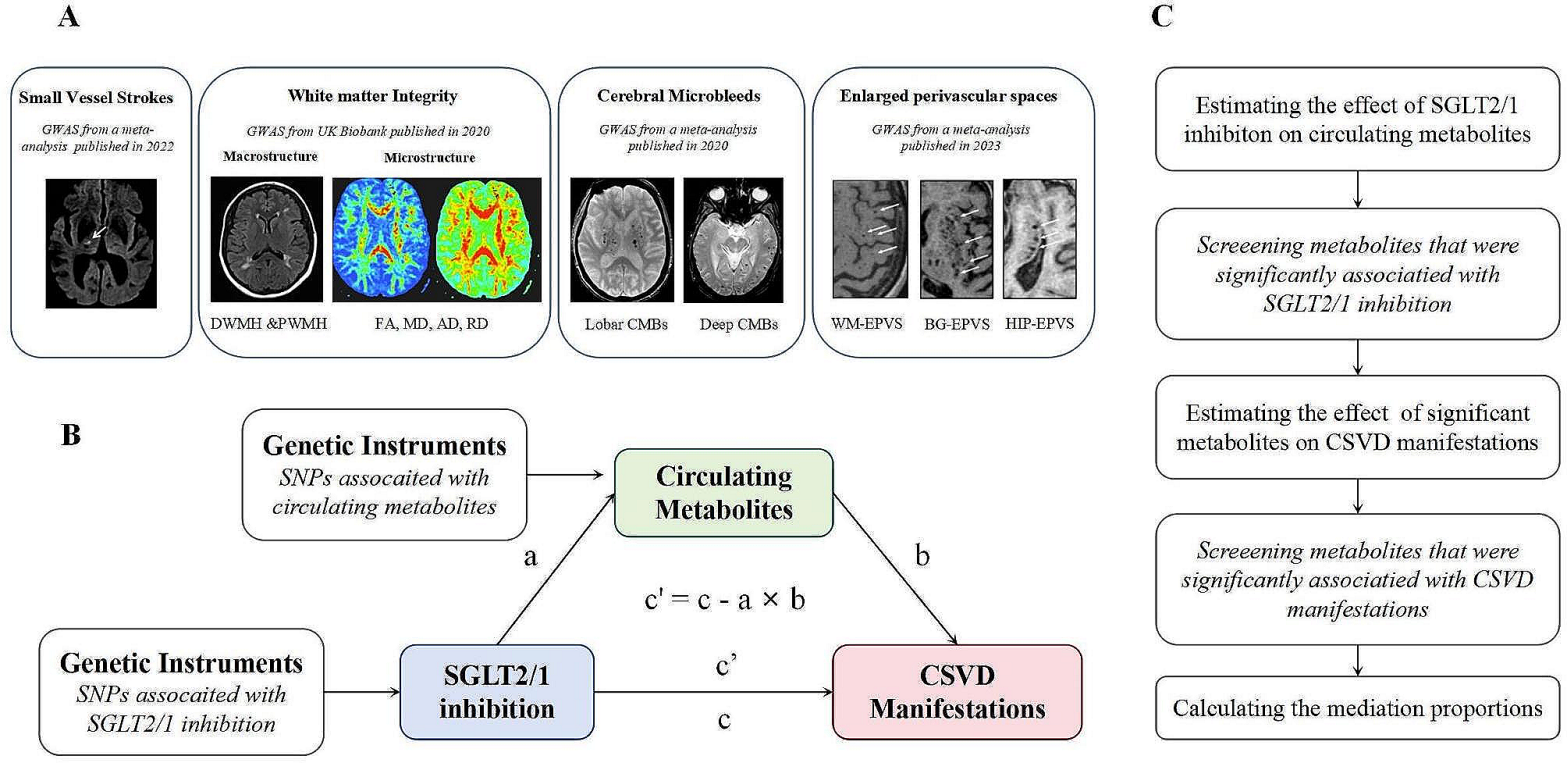
Overview of the study design. (A) The description of CSVD manifestations used as outcomes. (B) The framework of the two-step MR. (C) The flow diagram of conducting the two-step MR step by step, which involved the selection of circulating metabolites. *SNP, single nucleotide polymorphism; GWAS: Genome-Wide Association Studies; D/PWMH: deep/periventricular white matter hyperintensity; CMBs: cerebral microbleeds; (WM/BG/HIP)-EPVS: (white matter/basal ganglia/hippocampus)-enlarged perivascular spaces; SGLT: sodium-glucose cotransporter; CSVD: cerebral small vessel disease; FA: fractional anisotropy; MD: mean diffusivity; AD: axial diffusivity; RD: radial diffusivity

Figure 1 illustrates the study design. The mutual causation between SGLT1/2 inhibition and various CSVD manifestation (Fig. 1A) was assessed using a two-sample MR study. We further conducted a mediation analysis with a two-step MR design to explore whether circulating metabolites could mediate the causal pathway from SGLT1/2 inhibition to CSVD (Fig. 1B). This study followed the Strengthening the Reporting of Observational Studies in Epidemiology Using Mendelian Randomization (STROBE-MR) guideline [30].

### Genetic instruments for SGLT2 and SGLT1 inhibitors

According to the previously reported method, genetic variants for drug targets of SGLT1/2 inhibitors were selected using the summary data from the genome-wide association studies (GWAS) involving 344,182 non-diabetic individuals of European ancestry in the UK Biobank [31, 32]. To put it briefly, drug targets and encoding genes of SGLT1/2 inhibitors were identified. Genetic variations close to each gene that were both linked to the expression level of the relevant gene and the glycemic biomarker HbA1c were chosen. Following validation, each target’s genetic predictors were generated, with effects quantified as the HbA1c-lowering effect of the target.

### Genetic instruments for circulating metabolites

We utilized the most up-to-date and comprehensive GWAS datasets currently available for the human metabolome (Additional file 1) [33]. Based on the Canadian Longitudinal Study on Aging (CLSA) cohort, researchers analyzed data on 1,091 blood metabolites and 309 metabolite ratios by examining a total of 8,299 participants and approximately 15.4 million SNPs. The full GWAS summary statistics of the 1400 biomarkers were publicly available.

### CSVD manifestations

To evaluate the causal effect on the risk of small vessel stroke (SVS), we used the data from recently published cross-ancestry GWAS meta-analyses from the GIGASTROKE consortium [34]. As one of the clinically overt manifestations of CSVD and a subtype of ischemic stroke, SVS was identified through clinical evaluation and radiological confirmation [34]. SVS was discovered in 13,620 of 1,241,619 participants. The integrity of the white matter macrostructure was assessed using GWAS data of periventricular white matter hyperintensity (PWMH) and deep white matter hyperintensity (DWMH) volume, which were derived from the UK Biobank [35]. To reflect the integrity of white matter microstructure, GWAS data on average fractional anisotropy (FA), mean diffusivity (MD), axial diffusivity (AD), and radial diffusivity (RD) across 21 main white matter tracts were employed [36]. The statistical data on cerebral microbleeds (CMBs) were obtained from a meta-analysis of GWASes involving 11 population-based cohort studies and three case-control or case-only stroke research [37]. CMBs were detected in 3,172 of the 24,354 participants, of which 1,932 were lobar and 1,240 were deep. Up to 40,095 participants from 18 population-based cohorts in genome-wide association studies provided data on the burden of enlarged perivascular spaces (EPVS). The extensive EPVS burden was observed in the white matter (WM), basal ganglia (BG), and hippocampal (HIP) regions in 9,607 out of 39,822 participants, 9,189 out of 40,000 participants, and 9,339 out of 40,095 participants, respectively [38]. To minimize the risk of population stratification, we chose datasets that included nearly all European ancestry. The details could be found in Additional file 1.

### Selection of genetic instruments

We included single nucleotide polymorphisms (SNPs) with a genome-wide significant (*P* < 1 × 10^−5^). Then, these SNPs were clumped with a clumping window of 10,000 kb and a linkage disequilibrium (LD) level (r^2^ < 0.001). Estimated levels of LD from the 1000 Genomes Project based on European samples [39]. Palindromic and ambiguous SNPs were eliminated [40]. The strength of the instruments was judged by F statistics. We removed weak instrumental variables (F-statistics < 10) to avoid bias in MR analysis [41].

### Statistical analysis

#### Effect of SGLT1/2 inhibition on CSVD markers by the MR analysis

We used two-sample MR analyses to explore the causal relationships between SGLT1/2 inhibition and CSVD. The inverse–variance weighted (IVW) regression with a fixed effects model was selected as the primary method for causal inference [42]. Then, MR-Egger, weighted-median, weighted mode and simple mode methods were conducted to complement and enhance the reliability of the results. The Wald ratio for MR analysis was used when only one genetic instrument was available.

### Mediation MR analysis linking SGLT1/2 inhibition with CSVD via circulating metabolites

We further performed a mediation analysis using a two-step MR design to explore whether the level of certain metabolites could mediate the causal pathway from SGLT1/2 inhibition to respective manifestation of CSVD. We first estimated the effect of SGLT1/2 inhibition on circulating metabolites (a in Fig. 1B) using two-sample MR. Second, we evaluated the effect of those metabolites that showed statistically significant associations with SGLT1/2 inhibition on CSVD using two-sample MR (b in Fig. 1B). The total effect gained in previous MR analysis (c in Fig. 1B) can be decomposed into an indirect effect (through mediators, a × b in Fig. 1B) and a direct effect (without mediators, c’ in Fig. 1B) effect [43]. By dividing the indirect effect by the total effect, we could determine the percentage mediated by the mediating effect. Concurrently, the delta approach was utilized to compute 95% confidence intervals (CIs).

### Sensitivity analysis

With the aid of funnel plots and Cochran’s Q statistic, the heterogeneity between SNPs was evaluated [44]. The MR-PRESSO [45] and MR-Egger intercept [46] methods were used to identify horizontal pleiotropy. We eliminated any outliers that we found and reassessed the MR causal estimations. A random effects model, which is more resilient to weaker SNP exposure associations was used to evaluate the stability of the results if heterogeneity persisted after elimination of outliers. Ultimately, the impact of each SNP on the total causal estimates was verified using the leave-one-out analysis.

## Results

### Effect of SGLT2 and SGLT1 inhibition on CSVD

**Fig. 2:**
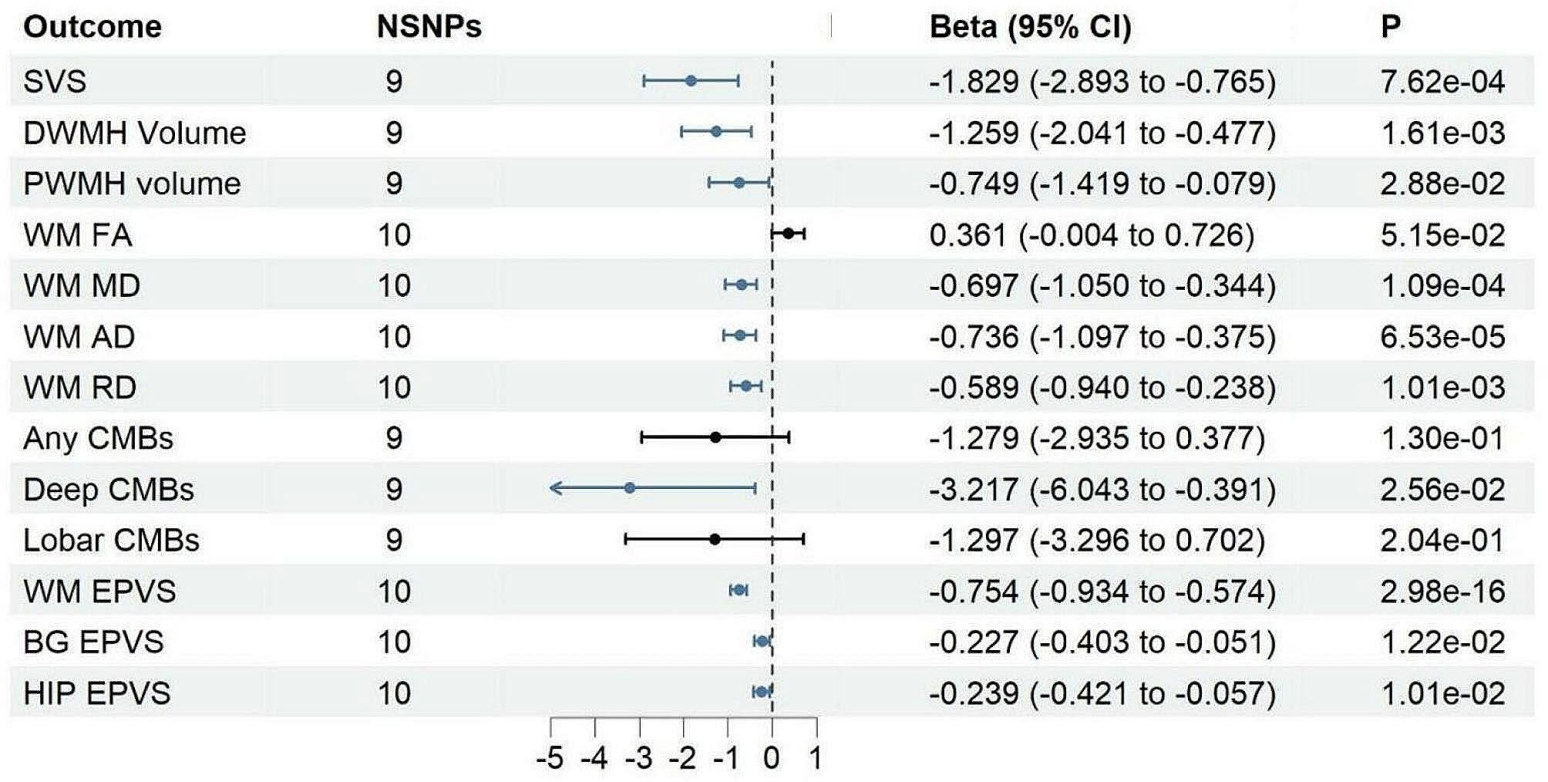
MR estimates (based on IVW) of the effect of SGLT2 inhibition on CSVD manifestations. *IVW: inverse–variance weighted; MR: Mendelian Randomization; SVS: small vessel stroke; SNP, single nucleotide polymorphism; D/PWMH: deep/periventricular white matter hyperintensity; CMBs: cerebral microbleeds; (WM/BG/HIP)-EPVS: (white matter/basal ganglia/hippocampus)-enlarged perivascular spaces; SGLT: sodium-glucose cotransporter; CSVD: cerebral small vessel disease; FA: fractional anisotropy; MD: mean diffusivity; AD: axial diffusivity; RD: radial diffusivity

**Fig. 3:**
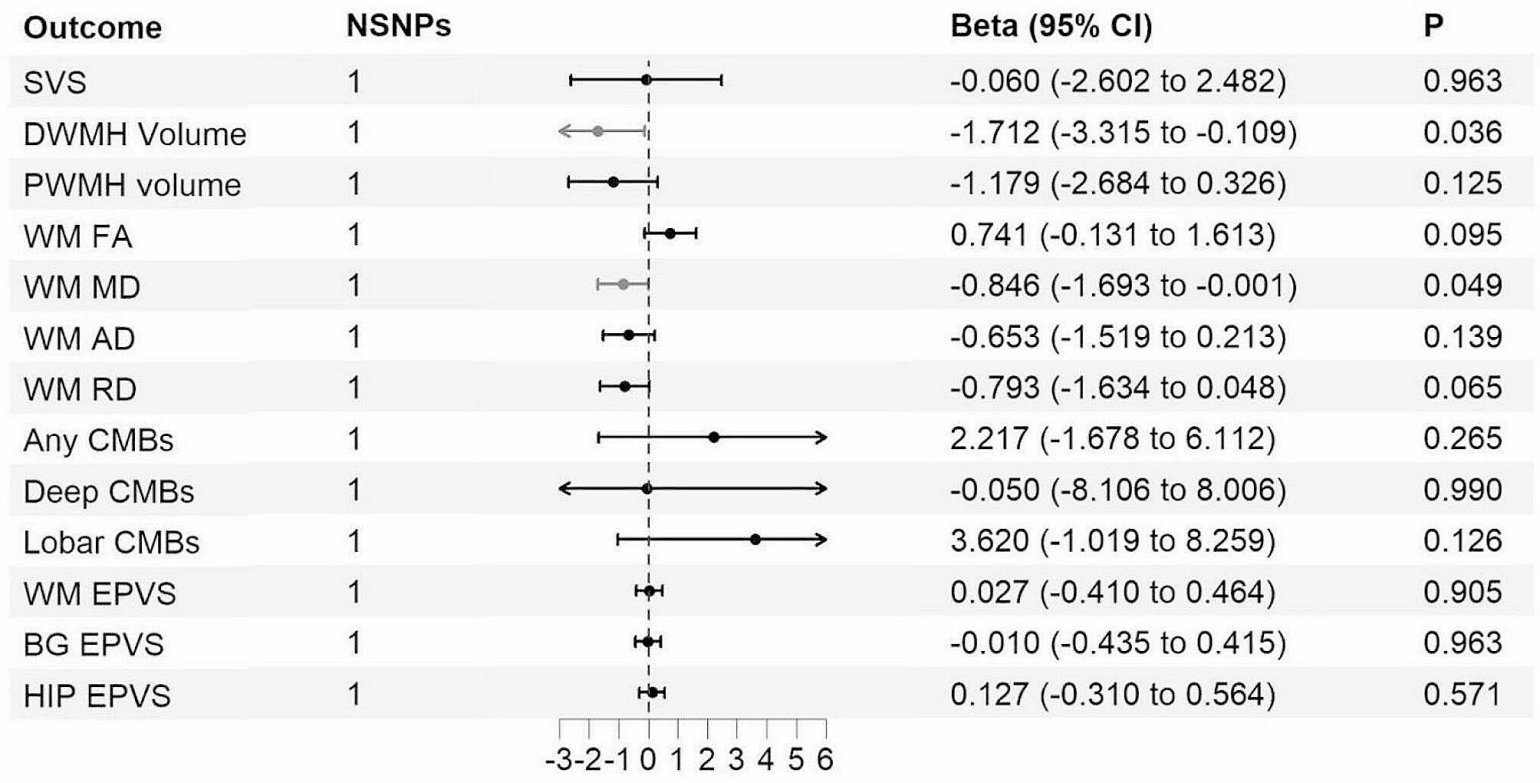
MR estimates (based on Wald ratio) of the effect of SGLT1 inhibition on CSVD manifestations. *MR: Mendelian Randomization; SVS: small vessel stroke; SNP, single nucleotide polymorphism; D/PWMH: deep/periventricular white matter hyperintensity; CMBs: cerebral microbleeds; (WM/BG/HIP)-EPVS: (white matter/basal ganglia/hippocampus)-enlarged perivascular spaces; SGLT: sodium-glucose cotransporter; CSVD: cerebral small vessel disease; FA: fractional anisotropy; MD: mean diffusivity; AD: axial diffusivity; RD: radial diffusivity

As shown in Additional file 2, ten distinct SNPs were chosen to serve as genetic tools for SGLT2 inhibition, and the F statistics for each SNP were more than 16. rs17683430 (F statistic 59), was used to instrument SGLT1 inhibition. As shown in Fig. 2 and Additional file 3, we found that SGLT2 inhibition was associated with a reduced risk of SVS [odds ratio (OR) = 0.161; 95% CI, 0.055−0.466, *P* < 0.001], deep CMBs (OR = 0.026; 95% CI, 0.002−0.676, *P* = 0.026) and better integrity of white matter structure, manifested as lower DWMH volume (Beta = –1.259; 95% CI, –2.041−0.477, *P* = 0.002), lower PWMH volume (Beta = 0.749; 95% CI, –1.420−-0.078, *P* = 0.029), decreased RD (Beta = –0.589; 95% CI, –0.940−–0.238, *P* = 0.001), decreased MD (Beta = –0.697; 95% CI, –1.050−–0.344, *P* = 1.09 × 10^−4^), and reduced AD (Beta = –0.736; 95% CI, –1.097−–0.375, *P* = 6.53 × 10^−5^). SGLT2 inhibition could further reduce the occurrence of severe EPVS located at withe matter (OR = 0.470; 95% CI, 0.393−0.564, *P* = 2.98 × 10^−16^), BG (OR = 0.797; 95% CI, 0.668−0.952, *P* = 0.012) and HIP regions (OR = 0.787, 95% CI, 0.656−0.945, *P* = 0.010). SGLT1 inhibition could protect white matter integrity, manifested as lower DWMH volume (Beta = –1.172; 95% CI, –3.315−-0.109, *P* = 0.036) and decreased MD (Beta = –0.846; 95% CI, –1.693−–0.001, *P* = 0.049) (Fig. 3 and Additional file 4). There was no heterogeneity or horizontal pleiotropy between instruments for analyzing the effect of SGLT1/2 inhibition on CSVD manifestations.

### Effect of SGLT1/2 inhibition on circulating metabolites and CSVD via the mediation MR analysis

We estimated the effect of SGLT1/2 inhibition on 1400 circulating metabolites and observed that 168 metabolites were significantly associated with SGLT2 inhibition [Bonferroni-corrected *P* < 3.57 × 10^−5^ (0.05/1400)] (Additional file 5) and that no metabolite was associated with SGLT1 inhibition. We further evaluated the effect of 168 circulating metabolites that were significantly associated with SGLT2 inhibition on SVS, deep CMBs, D/PWMH volume, EPVS located at different regions and white matter microstructures, including AD, RD, and MD (Additional file 6). The result showed that seven metabolites were associated with SVS, among which 4-acetamidobutanoate remained a substantial correlation after Bonferroni-correction [*P* < 2.98 × 10^−4^ (0.05/168)]. Two metabolites and four metaboliates were associated with PWMH volume and DWMH volume respectively, among which 7-methylxanthine had the strongest correlation (PWMH: *P* = 4.51 × 10^−4^, DWMH: *P* = 5.82 × 10^−3^), but it did not present a significant relationship after Bonferroni-correction. In terms of white matter microstructure, eight, six and seven metabolites were associated with MD, AD and RD of white matter, respectively. After Bonferroni-correction, the cholesterol to oleoyl-linoleoyl-glycerol (18:1 to 18:2) ratio (*P* = 1.91 × 10^−4^) was significantly associated with RD of white matter. For deep CMBs, seven metabolites showed a suggestively significant (*P* < 0.05, *P*_adj_ > 0.05) association. Seven, six and eleven metabolites were associated with BG-EPVS, WM-EPVS and HIP-EPVS respectively, but none showed a statistical association after Bonferroni-correction.

**Fig. 4:**
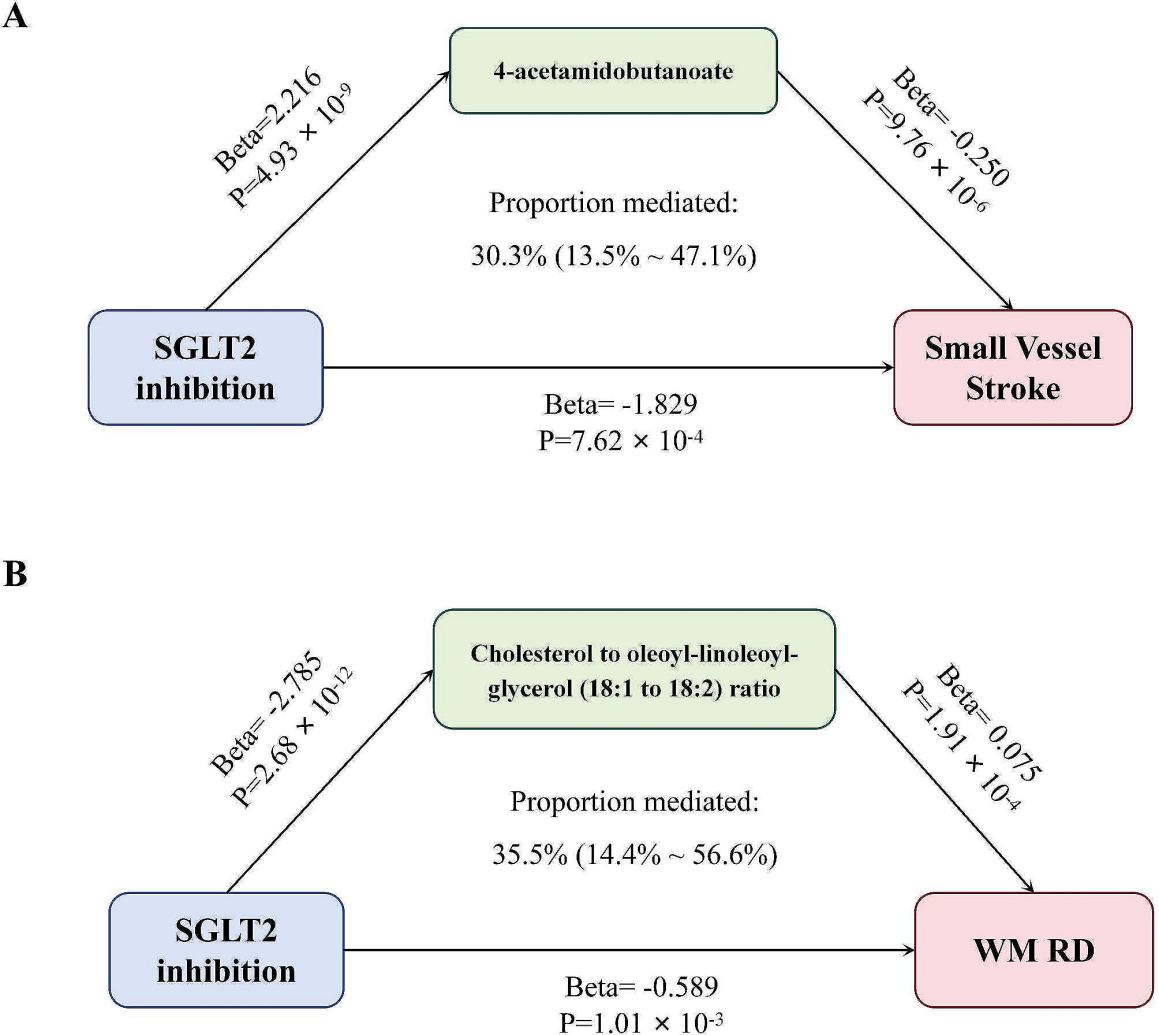
The potential causal evidence summarized from the MR analysis. (A) The mediation effects of 4-acetamidobutanoate on the association between SGLT2 inhibitors and SVS. (B) The mediation effects of cholesterol to oleoyl-linoleoyl-glycerol (18:1 to 18:2) ratio on the association between SGLT2 inhibitors and the RD of white matter. *MR: Mendelian Randomization; SVS: small vessel stroke; SNP, single nucleotide polymorphism; WM: white matter; SGLT: sodium-glucose cotransporter; RD: radial diffusivity

We observed an indirect effect of SGLT2 inhibition on SVS through 4-acetamidobutanoate, with a mediated proportion of 30.3% (95% CI, 13.5−47.1%, *P* = 4.19 × 10^−4^) of the total effect (Fig. 4A). The indirect effect of SGLT2 inhibition on RD of white matter through the cholesterol to oleoyl-linoleoyl-glycerol (18:1 to 18:2) ratio had a mediated proportion of 35.5% (95% CI, 14.4−56.6%, *P* = 9.96 × 10^−4^) (Fig. 4B). There was no evidence of heterogeneity and no horizontal pleiotropy among these associations.

## Discussion

### Principal findings

In the present study, we evaluated the associations between genetically predicted SGLT1/2 inhibition and CSVD and investigated the mediating role of circulating metabolites in the relationship. Our study indicated that the genetic variation for targets of SGLT2 inhibition was associated with a lower risk of SVS, deep CMBs and a lower burden of EPVS. Both SGLT2 and SGLT1 inhibition might lead to better integrity of white matter structure. The total concentration of 4-acetamidobutanoate and the cholesterol to oleoyl-linoleoyl-glycerol (18:1 to 18:2) ratio could partially mediate the effect of SGLT2 inhibition on SVS and RD of white matter, respectively.

### Association between SGLT1/2 inhibition and CSVD

Among studies investigating the effect of SGLT1/2 inhibition on cerebrovascular dysfunction, most research focused on the risk of stroke. Large clinical trials and meta-analyses have investigated the role of SGLT2 inhibition in fatal or non-fatal stroke and ischemic stroke. Intracerebral ventricular phlorizin [47, 48] and antisense SGLT1 mRNA treatment [49] have been indicated to decrease the size of the infarct. Additionally, studies demonstrated a relatively stronger protective effect of SGLT1/SGLT2 inhibition against stroke [50]. However, no study revealed the effects of SGLT1/SGLT2 inhibition on CSVD progression such as the occurrence of SVS and increased volume of WMH. The present study first reported the potential protective effect of SGLT2 and SGLT1 inhibition on CSVD. In line with our findings, numerous prior studies have demonstrated the preventive effect of SGLT2 and SGLT1 inhibitors on the pathological alterations linked to CSVD. The primary expression site of SGLT2 in the brain is microvasculature [51], which is closely related to the neurovascular unit (NVU), a fundamental biological system. As a key structural and functional component of the blood–brain barrier (BBB), NVU plays an important role in CSVD pathogenesis [52]. The NVU’s cell and myelin ultrastructural remodeling, including endothelial cells (ECs) and cortical matter aberration as well as the attenuation or loss of EC tight and adherent junctions of the BBB, could be avoided by suppressing SGLT2 expression with empagliflozin (EMP) [53]. Additionally, much evidence has suggested neuroinflammation as one of the main pathophysiological mechanisms of CSVD progression, with microglia serving as the catalyst for this process [54, 55]. Following EMP treatment, there was a decrease in the levels of tumor necrosis factor-alpha (TNF-α), interleukin-6 (IL-6), and monocyte chemoattractant protein-1 (MCP-1)—all of which were strongly linked to M1 microglia activation, which had a high capacity for phagocytic activity [56–58]. Hierro-Bujalance et al. also observed reduced microglia burden in the parenchyma of db/db and APP/PS1xdb/db mice treated with EMP [59]. The reduction of oxidative stress, as seen by lower levels of malondialdehyde, higher catalase activity, and higher glutathione, may also underly the neuroprotective effect of empagliflozin [60]. Like SGLT2, cerebral SGLT1 exists in neurons and ECs of small vessels [61, 62]. Under hypoxic conditions, ECs from small arteries express more SGLT1 [62, 63], which triggers microglia activation and causes vascular cognitive impairments in CSVD [19]. It also enhances the expression of MCP-1, IL-1β, TNF-α, and IL-6 in neurons and/or ECs [19]. Thus, SGLT1 gene deletion may protect against CSVD by inhibiting macrophage infiltration and microglia activation.

### Association between SGLT1/2 inhibition and circulating metabolites

The effects of SGLT2 inhibitors on metabolites have been frequently reported. Our results further emphasized the impact of SGLT2 inhibitors on the metabolism of amino acids, especially leucine and isoleucine, both of which belonged to branched-chain amino acids (BCAAs). Newgard et al. has reported the link between elevated plasma concentrations of BCAA and insulin resistance [64]. However, a recent study found that 20 g BCAA daily for four weeks could improve glucose metabolism [65]. Furthermore, an investigation revealed that an eight-week intake of BCAAs reduced liver fat accumulation [66]. Furuya et al. also detected higher plasma levels of valine and leucine after one-week administration of SGLT2 inhibitor [67]. Together with our results, these findings implied that BCAA improved insulin resistance rather than worsened it and might regulate the ameliorating effect of SGLT2 inhibitors. SGLT1 inhibitors were less likely to affect metabolite levels compared with SGLT2 inhibitors. Prior research has suggested that the positive effects of SGLT1 inhibitors may be attributed to that they can regulate inflammatory cytokines to prevent macrophage infiltration and microglia activation [19]. Consequently, the effect of SGLT1 inhibitors could not be fully captured by the metabolites included in the present investigation. The exact mechanism by which SGLT1 inhibitors preserved the integrity of white matter should be further investigated.

### Mediating role of circulating metabolites in the association between SGLT2 inhibition and CSVD manifestations

It was noteworthy that genetically predicted SGLT2 inhibition might lower the risk of SVS by modulating the level of 4-acetamidobutanoate. As a urea cycle product, 4-acetamidobutanoate was associated with the polyamine (PA) metabolism. The PA was secreted from intracellular compartments in various cerebral nervous system (CNS) traumas, such as focal cerebral ischemia in the ischemic cascade [68, 69]. In rat brain homogenates, it acted as free radical scavengers to lessen lipid peroxidation brought on by prooxidant agents like quinolinic acid, iron (Fe^2+^), and sodium nitroprusside [70, 71]. Due to the capacity of PA to raise nitric oxide bioavailability, lower oxidative stress, change structural variables, and promote autophagy, it might improve EC function [72]. 4-acetamidobutanoate was also reported to be a derivative of gamma-aminobutyric acid (GABA) [73], a crucial inhibitory neurotransmitter in the human CNS. The efficacy of hyperpolarization and chloride transport across the postsynaptic membrane could be enhanced by GABA neurotransmission. As demonstrated by the neuroprotection of GABA receptor agonists in animal stroke models, these actions could balance the harmful effects of glutamate during cerebral ischemia. GABA levels in the cerebro-spinal fluid (CSF) and plasma were shown to be lower in patients suffering from global cerebral ischemia and acute ischemic stroke [74].

The mediating role of the cholesterol to oleoyl-linoleoyl-glycerol (18:1 to 18:2) ratio in the connection between SGLT2 inhibition and white matter microstructure further highlighted the importance of cholesterol and its homeostasis in CSVD. Cholesterol played a pivotal role in the brain and brain diseases. The high cholesterol diet-induced hypercholesterolemia could cause microgliosis, astrogliosis and white matter inflammation [75]. Animal models of familial hypercholesterolemia also exhibited angiopathology associated with white matter injuries like BBB disruption [76, 77]. Numerous mechanisms, including intraluminal cell accumulations [78], reduced vasodilatory response [79], severe inflammation, and thrombosis [80, 81], connected hypercholesterolemia to the pathological alterations of small arteries and capillaries in CSVD. In addition to total cholesterol levels, the integrity of white matter has been shown to destroy cholesterol homeostasis, manifested as elevated low-density lipoprotein cholesterol (LDL-c) level and decreased high-density lipoprotein cholesterol (HDL-c) concentration [82]. Nevertheless, LDL-c, particularly small and dense LDL-c (sdLDL-c), was easily oxidized and slowly cleared, thus entering the artery wall to cause atherosclerosis [83], which was harmful to white matter, HDL-c maintains the integrity of white matter by regulating the function of vascular smooth muscle cells, improving EC function, and maintaining BBB integrity [84, 85]. Oleoyl-linoleoyl-glycerol (18:1 to 18:2) [DAG (36:3)] is a type of diacylglycerol. By stimulating the production and release of HDL-c, diacylglycerol was able to raise HDL-c levels, while at the same time lowering total cholesterol and LDL-c levels by speeding up the elimination of LDL-c and blocking the synthesis of cholesterol. Increased plasma levels of DAG (36:3) were associated with moderate to severe obstructive sleep apnea (OSA) [86], which was an independent risk factor for asymptomatic CSVD, particularly WMH and acute small subcortical infarcts [87].

### Strengths and limitations

As far as we know, this study stands out as the first to delve into the connection between SGLT2 and SGLT1 inhibition, circulating metabolites, and CSVD manifestations using MR analysis. Moreover, the genetic evidence supporting the possible mechanism of SGLT2 inhibition in preventing CSVD was presented. Still, some limitations merit consideration. To begin with, genetic variations that mimic SGLT2 inhibition revealed the lifelong effects of SGLT2 inhibitors; however, these effects may differ from that of SGLT2 inhibitors used for a short period. Besides, given that CSVD is an age-related and progressive disease, patients in various age groups or disease stages might respond differently to SGLT inhibition. As such, the direction rather than the amount of the probable causal influence can be better investigated in our study. Experiments and large-scale clinical trials based on different population such as very elderly population are warranted for further investigation. Second, as a complex disease, CSVD can present different types and distribution patterns of imaging markers. Although the most representative imaging markers were selected, we could not cover all CSVD manifestations. Moreover, there were overlapped samples in GWAS of SGLT1/2 inhibition and CSVD manifestations, which might cause a bias in the potential causal effect estimate in the case of the weak instruments [88]. Nonetheless, the genetic variations for SGLT1/2 inhibition were highly correlated with exposure, as shown by high F-statistics, suggesting the validity of our findings. Finally, because the data used in the present study were derived mainly from the European population, the extrapolation of the results to other ethnic populations should be considered with caution.

## Conclusions

In conclusion, our findings indicate that SGLT2 and SGLT1 may prevent CSVD development. The SGLT2 inhibition may lower the risk of SVS and improve the integrity of white matter microstructure by modulating the level of 4-acetamidobutanoate and cholesterol metabolism. These findings provide novel insights into the value of SGLT inhibition in preventing CSVD and might guide future mechanistic and clinical studies.

### Electronic supplementary material

Below is the link to the electronic supplementary material.

**Additional file 1:** **Table S1**. Detailed information for genome-wide association study (GWAS) statistics used in the present study.
** Additional file 2:** **Table S2**. Instrumental variables for SGLT2 and SGLT1 inhibition.
** Additional file 3:** **Table S3**. MR estimates of the effect of SGLT2 inhibition on CSVD manifestations.
** Additional file 4:** **Table S4**. MR estimates of the effect of SGLT1 inhibition on CSVD manifestations.
** Additional file 5: Table S5**. Significant (p<0.05) MR estimates of the effect of SGLT2 inhibition on 1400 metabolites. 
** Additional file 6:**  **Table S6**. The effects of SGLT2 inhibition on circulating metabolites and the effects of metabolites on CSVD manifestations.

## Data Availability

All GWAS summary statistics used for the included MR analyses could be found in Supplemental data. R scripts for these analyses could be shared upon request (lvyanchenfudan@163.com).

## Abbreviations

AD: Axial diffusivity
BBB: Blood–brain barrier
BCAA: Branched chain amino acids
CIs: Confidence intervals
CLSA: Canadian Longitudinal Study on Aging
CMBs: Cerebral microbleeds
CNS: Cerebral nervous system
CSF: Cerebro-spinal fluid
CSVD: Cerebral small vessel disease
DAG: Diacylglycerol
D/PWMH: Deep/periventricular white matter hyperintensity
(WM/BG/HIP)-EPVS: (white matter/basal ganglia/hippocampus)-enlarged perivascular spaces
EC: Endothelial cell
EMP: Empagliflozin
FA: Fractional anisotropy
GABA: Gamma-aminobutyric acid
GWAS: Genome-Wide Association Studies
HbA1C: Glycated hemoglobin level
HDL-c: High-density lipoprotein cholesterol
IL: Interleukin
IVW: Inverse–variance weighted
LD: Linkage disequilibrium
LDL-c: Low-density lipoprotein cholesterol
MCP-1: Monocyte chemoattractant protein-1
MD: Mean diffusivity
MR: Mendelian Randomization
NYU: Neurovascular unit
OR: Odds ratio
OSA: Obstructive sleep apnea
PA: Polyamine
RD: Radial diffusivityradial diffusivity
sdLDL-C: Small and dense low-density lipoprotein cholesterol
SGLT: Sodium-glucose cotransporter
SNP: Single nucleotide polymorphism
STROBE-MR: Strengthening the Reporting of Observational Studies in Epidemiology Using Mendelian Randomization
SVS: Small vessel stroke
TNF: Tumor necrosis factor
WM: White matter
