# Supplementary material for: SGLT1 and SGLT2 inhibition, circulating metabolites, and cerebral small vessel disease: a mediation Mendelian Randomization study
Source: Cardiovasc Diabetol. 2024 May 7;23:157. doi: 10.1186/s12933-024-02255-6 (PMC11077823; doi:10.1186/s12933-024-02255-6)
Supplement: Supplementary file 1 — Additional file 1: Table S1. Detailed information for genome-wide association study (GWAS) statistics used in the present study. Additional file 2: Table S2. Instrumental variables for SGLT2 and SGLT1 inhibition. Additional file 3: Table S3. MR estimates of the effect of SGLT2 inhibition on CSVD manifestations. Additional file 4: Table S4. MR estimates of the effect of SGLT1 inhibition on CSVD manifestations. Additional file 5: Table S5. Significant (p<0.05) MR estimates of the effect of SGLT2 inhibition on 1400 metabolites. Additional file 6: Table S6. The effects of SGLT2 inhibition on circulating metabolites and the effects of metabolites on CSVD manifestations. [file 12933_2024_2255_MOESM1_ESM.docx]

**SGLT1 and SGLT2 inhibition, circulating metabolites, and cerebral small vessel disease: a mediation Mendelian Randomization study**

**Supplementary Tables**

Supplementary Table 1. Detailed information for genome-wide association study (GWAS) statistics used in the present study

Supplementary Table 2. Instrumental variables for SGLT2 and SGLT1 inhibition

Supplementary Table 3. MR estimates of the effect of SGLT2 inhibition on CSVD manifestations

Supplementary Table 4. MR estimates of the effect of SGLT1 inhibition on CSVD manifestations

Supplementary Table 5. Significant (p<0.05) MR estimates of the effect of SGLT2 inhibition on 1400 metabolites

Supplementary Table 1. Detailed information for genome-wide association study (GWAS) statistics used in the present study

| **Phenotype** | **Consortium** | **Sample size** | **Ancestry** | **Year** | **IEU Open GWAS ID or study reference** |
| --- | --- | --- | --- | --- | --- |
| SGLT2 inhibition | UK Biobank | 344,182 | European | 2018 | ukb-d-30750_irnt |
| Circulating metabolites | Canadian Longitudinal Study on Aging (CLSA) cohort | 8299 | European | 2023 | Nat Genet. 2023Jan;55(1):44-53. |
| Small vessel stroke | GIGASTROKE consortium | 1241619 | cross-ancestry | 2022 | Nature. 2022 Nov;611(7934):115-123. |
| DWMH &PWMH volume | UK Biobank | 8428 | cross-ancestry | 2018 | Nature. 2018 Oct;562(7726):210-216. |
| FA, MD, AD, RD | UK Biobank | 43802 | cross-ancestry | 2021 | Science. 2021 Jun 18;372(6548):eabf3736. |
| CMBs (Any, deep, lobar) | the Cohorts of Heart and Aging Research in Genomic Epidemiology (CHARGE) consortium, UK Biobank, Alzheimer's Disease Neuroimaging Initiative (ADNI) database, Massachusetts General Hospital Genes Affecting Stroke Risk and Outcomes Study (MGH-GASROS), Clinical Relevance of Microbleeds in Stroke due to Atrial Fibrillation (CROMIS-2 AF) | 25862 | cross-ancestry | 2020 | Neurology. 2020 Dec 15;95(24):e3331-e3343. |
| EPVS | Cohorts for Heart and Aging Research in Genomic Epidemiology (CHARGE) consortium, UK Biobank, | 40095 | cross-ancestry | 2023 | Nat Med. 2023 Apr;29(4):950-962. |

Supplementary Table 2. Instrumental variables for SGLT2 and SGLT1 inhibition

| **SNP** | **effect_allele** | **other_allele** | **MAF** | **beta** | **se** | **P** | **F-statistics** |
| --- | --- | --- | --- | --- | --- | --- | --- |
| SGLT2 inhibition | | | | | | | |
| rs111510548 | C | T | 0.103 | 0.015 | 0.004 | 6.69E-05 | 16 |
| rs8057207 | T | C | 0.362 | 0.013 | 0.002 | 4.55E-08 | 30 |
| rs9926717 | G | A | 0.284 | 0.011 | 0.003 | 9.61E-06 | 20 |
| rs116943658 | A | G | 0.33 | 0.013 | 0.002 | 3.01E-07 | 26 |
| rs13334492 | A | G | 0.442 | 0.011 | 0.002 | 7.57E-07 | 24 |
| rs2070896 | C | T | 0.375 | 0.017 | 0.002 | 1.70E-12 | 50 |
| rs28641848 | T | C | 0.279 | 0.011 | 0.003 | 1.21E-05 | 19 |
| rs28692853 | A | C | 0.507 | 0.015 | 0.002 | 2.78E-10 | 40 |
| rs67464975 | T | C | 0.47 | 0.012 | 0.002 | 4.03E-07 | 26 |
| rs8050328 | G | T | 0.364 | 0.016 | 0.002 | 1.09E-11 | 46 |
| SGLT2 inhibition | | | | | | | |
| rs17683430 | A | G | 0.08 | -0.03 | 0.004 | 6.24E-16 | 59 |

Supplementary Table 3. MR estimates of the effect of SGLT2 inhibition on CSVD manifestations

| Outcome | Method | Number of SNPs | beta | se | p value | IVW Q test P | Egger intercept P | p value of MR-PRESSO Global test | p value of MR-PRESSO Distortion test |
| --- | --- | --- | --- | --- | --- | --- | --- | --- | --- |
| SVS | MR Egger | 9 | 0.653 | 3.327 | 8.50E-01 | 0.941 | 0.474 | 0.947 | \ |
|  | Weighted median | 9 | -1.380 | 0.680 | 4.24E-02 |  |  |  |  |
|  | Inverse variance weighted | 9 | -1.829 | 0.543 | 7.62E-04 |  |  |  |  |
|  | Simple mode | 9 | -1.468 | 1.050 | 2.00E-01 |  |  |  |  |
|  | Weighted mode | 9 | -1.363 | 0.856 | 1.50E-01 |  |  |  |  |
|  | MR-PRESSO(Outlier-corrected) | \ | \ | \ | \ |  |  |  |  |
| DWMH Volume | MR Egger | 9 | 3.500 | 2.217 | 1.58E-01 | 0.296 | 0.066 | 0.325 | \ |
|  | Weighted median | 9 | -1.279 | 0.512 | 1.26E-02 |  |  |  |  |
|  | Inverse variance weighted | 9 | -1.259 | 0.399 | 1.61E-03 |  |  |  |  |
|  | Simple mode | 9 | -2.050 | 0.949 | 6.28E-02 |  |  |  |  |
|  | Weighted mode | 9 | -1.727 | 0.816 | 6.73E-02 |  |  |  |  |
|  | MR-PRESSO(Outlier-corrected) | \ | \ | \ | \ |  |  |  |  |
| PWMH volume | MR Egger | 9 | 2.889 | 2.081 | 2.08E-01 | 0.629 | 0.120 | 0.601 | \ |
|  | Weighted median | 9 | -0.862 | 0.464 | 6.29E-02 |  |  |  |  |
|  | Inverse variance weighted | 9 | -0.749 | 0.342 | 2.88E-02 |  |  |  |  |
|  | Simple mode | 9 | -1.268 | 0.743 | 1.26E-01 |  |  |  |  |
|  | Weighted mode | 9 | -0.971 | 0.681 | 1.92E-01 |  |  |  |  |
|  | MR-PRESSO(Outlier-corrected) | \ | \ | \ | \ |  |  |  |  |
| FA | MR Egger | 10 | -0.341 | 1.188 | 7.81E-01 | 0.973 | 0.566 | 0.964 | \ |
|  | Weighted median | 10 | 0.209 | 0.250 | 4.03E-01 |  |  |  |  |
|  | Inverse variance weighted | 10 | 0.361 | 0.186 | 5.15E-02 |  |  |  |  |
|  | Simple mode | 10 | 0.124 | 0.361 | 7.39E-01 |  |  |  |  |
|  | Weighted mode | 10 | 0.134 | 0.339 | 7.01E-01 |  |  |  |  |
|  | MR-PRESSO(Outlier-corrected) | \ | \ | \ | \ |  |  |  |  |
| MD | MR Egger | 10 | 0.881 | 1.153 | 4.67E-01 | 0.920 | 0.203 | 0.936 | \ |
|  | Weighted median | 10 | -0.695 | 0.234 | 2.99E-03 |  |  |  |  |
|  | Inverse variance weighted | 10 | -0.697 | 0.180 | 1.09E-04 |  |  |  |  |
|  | Simple mode | 10 | -0.812 | 0.380 | 6.15E-02 |  |  |  |  |
|  | Weighted mode | 10 | -0.686 | 0.334 | 6.99E-02 |  |  |  |  |
|  | MR-PRESSO(Outlier-corrected) | \ | \ | \ | \ |  |  |  |  |
| AD | MR Egger | 10 | 0.912 | 1.180 | 4.62E-01 | 0.925 | 0.195 | 0.948 | \ |
|  | Weighted median | 10 | -0.737 | 0.234 | 1.60E-03 |  |  |  |  |
|  | Inverse variance weighted | 10 | -0.736 | 0.184 | 6.53E-05 |  |  |  |  |
|  | Simple mode | 10 | -0.945 | 0.368 | 3.03E-02 |  |  |  |  |
|  | Weighted mode | 10 | -0.720 | 0.325 | 5.37E-02 |  |  |  |  |
|  | MR-PRESSO(Outlier-corrected) | \ | \ | \ | \ |  |  |  |  |
| RD | MR Egger | 10 | 0.707 | 1.147 | 5.55E-01 | 0.949 | 0.285 | 0.951 | \ |
|  | Weighted median | 10 | -0.570 | 0.243 | 1.88E-02 |  |  |  |  |
|  | Inverse variance weighted | 10 | -0.589 | 0.179 | 1.01E-03 |  |  |  |  |
|  | Simple mode | 10 | -0.582 | 0.352 | 1.33E-01 |  |  |  |  |
|  | Weighted mode | 10 | -0.541 | 0.320 | 1.25E-01 |  |  |  |  |
|  | MR-PRESSO(Outlier-corrected) | \ | \ | \ | \ |  |  |  |  |
| Any CMBs | MR Egger | 9 | 4.606 | 5.122 | 3.98E-01 | 0.554 | 0.282 | 0.591 | \ |
|  | Weighted median | 9 | -0.937 | 1.156 | 4.18E-01 |  |  |  |  |
|  | Inverse variance weighted | 9 | -1.279 | 0.845 | 1.30E-01 |  |  |  |  |
|  | Simple mode | 9 | 1.338 | 1.605 | 4.29E-01 |  |  |  |  |
|  | Weighted mode | 9 | -0.064 | 1.415 | 9.65E-01 |  |  |  |  |
|  | MR-PRESSO(Outlier-corrected) | \ | \ | \ | \ |  |  |  |  |
| Deep CMBs | MR Egger | 9 | 14.19 | 8.735 | 1.48E-01 | 0.491 | 0.083 | 0.494 | \ |
|  | Weighted median | 9 | -3.480 | 2.056 | 9.05E-02 |  |  |  |  |
|  | Inverse variance weighted | 9 | -3.217 | 1.442 | 2.56E-02 |  |  |  |  |
|  | Simple mode | 9 | -6.298 | 3.693 | 1.27E-01 |  |  |  |  |
|  | Weighted mode | 9 | -6.350 | 3.082 | 7.34E-02 |  |  |  |  |
|  | MR-PRESSO(Outlier-corrected) | \ | \ | \ | \ |  |  |  |  |
| Lobar CMBs | MR Egger | 9 | -1.142 | 6.180 | 8.59E-01 | 0.591 | 0.980 | 0.694 |  |
|  | Weighted median | 9 | -0.823 | 1.282 | 5.21E-01 |  |  |  |  |
|  | Inverse variance weighted | 9 | -1.297 | 1.020 | 2.04E-01 |  |  |  |  |
|  | Simple mode | 9 | -0.792 | 1.514 | 6.15E-01 |  |  |  |  |
|  | Weighted mode | 9 | -0.725 | 1.460 | 6.33E-01 |  |  |  |  |
|  | MR-PRESSO(Outlier-corrected) | \ | \ | \ | \ |  |  |  |  |
| WM_EPVS | MR Egger | 10 | -0.036 | 0.591 | 9.53E-01 | 0.833 | 0.253 | 0.912 |  |
|  | Weighted median | 10 | -0.721 | 0.123 | 4.60E-09 |  |  |  |  |
|  | Inverse variance weighted | 10 | -0.754 | 0.092 | 2.98E-16 |  |  |  |  |
|  | Simple mode | 10 | -0.765 | 0.187 | 2.70E-03 |  |  |  |  |
|  | Weighted mode | 10 | -0.730 | 0.174 | 2.30E-03 |  |  |  |  |
|  | MR-PRESSO(Outlier-corrected) | \ | \ | \ | \ |  |  |  |  |
| BG_EPVS | MR Egger | 10 | -0.231 | 0.576 | 6.99E-01 | 0.999 | 0.994 | 0.999 |  |
|  | Weighted median | 10 | -0.246 | 0.108 | 2.23E-02 |  |  |  |  |
|  | Inverse variance weighted | 10 | -0.227 | 0.090 | 1.22E-02 |  |  |  |  |
|  | Simple mode | 10 | -0.264 | 0.169 | 1.53E-01 |  |  |  |  |
|  | Weighted mode | 10 | -0.255 | 0.155 | 1.34E-01 |  |  |  |  |
|  | MR-PRESSO(Outlier-corrected) | \ | \ | \ | \ |  |  |  |  |
| HIP_EPVS | MR Egger | 10 | -0.620 | 0.593 | 3.27E-01 | 0.600 | 0.534 | 0.634 |  |
|  | Weighted median | 10 | -0.258 | 0.129 | 4.59E-02 |  |  |  |  |
|  | Inverse variance weighted | 10 | -0.239 | 0.093 | 1.01E-02 |  |  |  |  |
|  | Simple mode | 10 | -0.207 | 0.190 | 3.05E-01 |  |  |  |  |
|  | Weighted mode | 10 | -0.287 | 0.181 | 1.47E-01 |  |  |  |  |
|  | MR-PRESSO(Outlier-corrected) | \ | \ | \ | \ |  |  |  |  |

Supplementary Table 4. MR estimates of the effect of SGLT1 inhibition on CSVD manifestations

| Outcome | Method | Number of SNPs | beta | se | p value | IVW Q test P | Egger intercept P | p value of MR-PRESSO Global test | p value of MR-PRESSO Distortion test |
| --- | --- | --- | --- | --- | --- | --- | --- | --- | --- |
| SVS | Wald Ratio | 1 | -0.060 | 1.297 | 9.63E-01 | \ | \ | \ | \ |
| DWMH Volume | Wald Ratio | 1 | -1.712 | 0.818 | 3.64E-02 | \ | \ | \ | \ |
| PWMH Volume | Wald Ratio | 1 | -1.179 | 0.768 | 1.25E-01 | \ | \ | \ | \ |
| WM_FA | Wald Ratio | 1 | 0.741 | 0.445 | 9.54E-02 | \ | \ | \ | \ |
| WM_MD | Wald Ratio | 1 | -0.846 | 0.431 | 4.99E-02 | \ | \ | \ | \ |
| WM_AD | Wald Ratio | 1 | -0.653 | 0.442 | 1.39E-01 | \ | \ | \ | \ |
| WM_RD | Wald Ratio | 1 | -0.793 | 0.429 | 6.45E-02 | \ | \ | \ | \ |
| Any_CMBs | Wald Ratio | 1 | 2.217 | 1.987 | 2.65E-01 | \ | \ | \ | \ |
| Deep_CMBs | Wald Ratio | 1 | -0.050 | 4.110 | 9.90E-01 | \ | \ | \ | \ |
| Lobar_CMBs | Wald Ratio | 1 | 3.620 | 2.367 | 1.26E-01 | \ | \ | \ | \ |
| WM_EPVS | Wald Ratio | 1 | 0.027 | 0.223 | 9.05E-01 | \ | \ | \ | \ |
| BG_EPVS | Wald Ratio | 1 | -0.010 | 0.217 | 9.63E-01 | \ | \ | \ | \ |
| HIP_EPVS | Wald Ratio | 1 | 0.127 | 0.223 | 5.71E-01 | \ | \ | \ | \ |

Supplementary Table 5. Significant (p<0.05) MR estimates of the effect of SGLT2 inhibition on 1400 metabolites

| trait | beta | se | pvalue | Q_pval | pleiotropy | SUPER_PATHWAY | SUB_PATHWAY | HMDB | HMDB_curated | KEGG |
| --- | --- | --- | --- | --- | --- | --- | --- | --- | --- | --- |
| N,N-dimethylalanine levels | 1.923 | 0.401 | 1.66E-06 | 0.632 | 0.374 | Amino Acid | Alanine and Aspartate Metabolism |  | 0 |  |
| Creatine levels | -1.634 | 0.365 | 7.57E-06 | 0.950 | 0.320 | Amino Acid | Creatine Metabolism | HMDB0000064 | HMDB0000064 | C00300 |
| Guanidinoacetate levels | 2.311 | 0.381 | 1.27E-09 | 0.951 | 0.447 | Amino Acid | Creatine Metabolism | HMDB0000128 | HMDB0000128 | C00581 |
| Cysteinylglycine levels | 1.920 | 0.394 | 1.10E-06 | 0.987 | 0.247 | Amino Acid | Glutathione Metabolism | HMDB0000078 | HMDB0000078 | C01419 |
| N-acetylglycine levels | 1.735 | 0.387 | 7.57E-06 | 0.901 | 0.318 | Amino Acid | Glycine, Serine and Threonine Metabolism | HMDB0000532 | HMDB0000532 |  |
| 1-methyl-4-imidazoleacetate levels | -2.593 | 0.428 | 1.39E-09 | 0.254 | 0.097 | Amino Acid | Histidine Metabolism | HMDB0002820 | HMDB0002820 | C05828 |
| Hydantoin-5-propionate levels | -2.278 | 0.398 | 1.05E-08 | 0.929 | 0.384 | Amino Acid | Histidine Metabolism | HMDB0001212 | HMDB0001212 | C05565 |
| N-acetylisoleucine levels | 1.837 | 0.423 | 1.44E-05 | 0.621 | 0.090 | Amino Acid | Leucine, Isoleucine and Valine Metabolism | HMDB0061684 | HMDB0061684 |  |
| Beta-hydroxyisovaleroylcarnitine levels | 2.033 | 0.393 | 2.27E-07 | 0.967 | 0.457 | Amino Acid | Leucine, Isoleucine and Valine Metabolism |  | 0 |  |
| N-lactoyl isoleucine levels | 1.981 | 0.405 | 1.03E-06 | 0.997 | 0.646 | Amino Acid | Leucine, Isoleucine and Valine Metabolism | HMDB0062180 | HMDB0062180 |  |
| N-acetyl leucine levels | 1.744 | 0.401 | 1.34E-05 | 0.978 | 0.344 | Amino Acid | Leucine, Isoleucine and Valine Metabolism | HMDB0011756 | HMDB0011756 | C02710 |
| N-acetylleucine levels | 1.759 | 0.405 | 1.38E-05 | 0.974 | 0.220 | Amino Acid | Leucine, Isoleucine and Valine Metabolism | HMDB0011756 | HMDB0011756 | C02710 |
| Leucine levels | 2.008 | 0.388 | 2.23E-07 | 0.934 | 0.785 | Amino Acid | Leucine, Isoleucine and Valine Metabolism | HMDB0000687 | HMDB0000687 | C00123 |
| Isoleucine levels | 2.666 | 0.392 | 1.05E-11 | 0.664 | 0.729 | Amino Acid | Leucine, Isoleucine and Valine Metabolism | HMDB0000172 | HMDB0000172 | C06418,C00407,C16424 |
| 6-oxopiperidine-2-carboxylate levels | -2.031 | 0.404 | 5.00E-07 | 0.973 | 0.408 | Amino Acid | Lysine Metabolism | HMDB0061705 | HMDB0061705 |  |
| Hydroxy-N6,N6,N6-trimethyllysine levels | -1.976 | 0.382 | 2.35E-07 | 0.828 | 0.218 | Amino Acid | Lysine Metabolism |  | 0 |  |
| Lysine levels | 1.674 | 0.399 | 2.70E-05 | 0.979 | 0.850 | Amino Acid | Lysine Metabolism | HMDB0003405 | HMDB0003405 | C00739,C00047 |
| N6,N6-dimethyllysine levels | -1.936 | 0.398 | 1.17E-06 | 0.827 | 0.240 | Amino Acid | Lysine Metabolism | HMDB0013287 | HMDB0013287 | C05545 |
| N2-acetyl,N6,N6-dimethyllysine levels | -2.237 | 0.406 | 3.64E-08 | 0.736 | 0.583 | Amino Acid | Lysine Metabolism |  | 0 |  |
| Cysteine s-sulfate levels | 2.167 | 0.392 | 3.34E-08 | 0.569 | 0.141 | Amino Acid | Methionine, Cysteine, SAM and Taurine Metabolism | HMDB0000731 | HMDB0000731 | C05824 |
| S-methylcysteine levels | 1.839 | 0.398 | 3.74E-06 | 0.831 | 0.152 | Amino Acid | Methionine, Cysteine, SAM and Taurine Metabolism | HMDB0002108 | HMDB0002108 |  |
| N-succinyl-phenylalanine levels | -2.774 | 0.460 | 1.60E-09 | 0.699 | 0.143 | Amino Acid | Phenylalanine Metabolism |  | 0 |  |
| N-acetyl-isoputreanine levels | 2.870 | 0.389 | 1.61E-13 | 0.979 | 0.318 | Amino Acid | Polyamine Metabolism |  | HMDB0094713 |  |
| 4-acetamidobutanoate levels | 2.216 | 0.379 | 4.93E-09 | 0.966 | 0.357 | Amino Acid | Polyamine Metabolism | HMDB0003681 | HMDB0003681 | C02946 |
| 3-indoxyl sulfate levels | -2.041 | 0.395 | 2.35E-07 | 0.686 | 0.375 | Amino Acid | Tryptophan Metabolism | HMDB0000682 | HMDB0000682 |  |
| Indoleacetylglutamine levels | -2.389 | 0.449 | 1.02E-07 | 0.575 | 0.101 | Amino Acid | Tryptophan Metabolism | HMDB0013240 | HMDB0013240 |  |
| 5-hydroxyindole sulfate levels | -1.956 | 0.464 | 2.50E-05 | 0.729 | 0.956 | Amino Acid | Tryptophan Metabolism |  | 0 |  |
| 6-bromotryptophan levels | 3.395 | 0.401 | 2.66E-17 | 0.824 | 0.590 | Amino Acid | Tryptophan Metabolism |  | 0 |  |
| Serotonin levels | -2.088 | 0.422 | 7.47E-07 | 0.944 | 0.979 | Amino Acid | Tryptophan Metabolism | HMDB0000259 | HMDB0000259 | C00780 |
| Gentisate levels | 1.709 | 0.399 | 1.80E-05 | 0.987 | 0.907 | Amino Acid | Tyrosine Metabolism | HMDB0000152 | HMDB0000152 | C00628 |
| Phenol sulfate levels | -2.063 | 0.403 | 3.12E-07 | 0.858 | 0.551 | Amino Acid | Tyrosine Metabolism | HMDB0060015 | HMDB0060015 | C02180 |
| 4-methoxyphenol sulfate levels | -1.749 | 0.415 | 2.48E-05 | 0.989 | 0.586 | Amino Acid | Tyrosine Metabolism |  | 0 |  |
| 4-hydroxyphenylpyruvate levels | -2.652 | 0.410 | 9.94E-11 | 0.909 | 0.253 | Amino Acid | Tyrosine Metabolism | HMDB0000707 | HMDB0000707 | C01179 |
| Urea levels | -1.916 | 0.385 | 6.33E-07 | 0.673 | 0.284 | Amino Acid | Urea cycle; Arginine and Proline Metabolism | HMDB0000294 | HMDB0000294 | C00086 |
| N-acetylcitrulline levels | -2.194 | 0.421 | 1.89E-07 | 0.682 | 0.187 | Amino Acid | Urea cycle; Arginine and Proline Metabolism | HMDB0000856 | HMDB0000856 | C15532,C02851 |
| Mannitol/sorbitol levels | -1.760 | 0.397 | 9.08E-06 | 0.991 | 0.569 | Carbohydrate | Fructose, Mannose and Galactose Metabolism | HMDB0000247,HMDB0000765 | HMDB0000247,HMDB0000765 | C00392,C00794 |
| Maltose levels in coronary artery disease | -3.625 | 0.454 | 1.33E-15 | 0.412 | 0.696 | Carbohydrate | Glycogen Metabolism | HMDB0000163 | HMDB0000163 | C00208 |
| Succinylcarnitine levels | -1.818 | 0.389 | 3.04E-06 | 0.650 | 0.784 | Energy | TCA Cycle | HMDB0061717 | HMDB0061717 |  |
| Cortisone levels | 2.531 | 0.389 | 7.86E-11 | 0.873 | 0.340 | Lipid | Corticosteroids | HMDB0002802 | HMDB0002802 | C00762 |
| Cortisol levels (plasma) | 1.814 | 0.395 | 4.47E-06 | 0.899 | 0.490 | Lipid | Corticosteroids | HMDB0000063 | HMDB0000063 | C00735 |
| Oleoyl-linoleoyl-glycerol (18:1/18:2) [2] levels | -2.317 | 0.404 | 9.53E-09 | 0.872 | 0.931 | Lipid | Diacylglycerol | HMDB0007219 | HMDB0007219 |  |
| Linoleoyl-arachidonoyl-glycerol (18:2/20:4) [1] levels | -2.628 | 0.413 | 2.06E-10 | 0.998 | 0.696 | Lipid | Diacylglycerol | HMDB0007257 | HMDB0007257 |  |
| N-oleoyltaurine levels | 1.984 | 0.415 | 1.72E-06 | 0.931 | 0.258 | Lipid | Endocannabinoid |  | 0 |  |
| Oleoyl ethanolamide levels | 1.988 | 0.393 | 4.24E-07 | 0.970 | 0.245 | Lipid | Endocannabinoid | HMDB0002088 | HMDB0002088 |  |
| Stearoylcarnitine levels | 3.062 | 0.396 | 9.81E-15 | 0.921 | 0.299 | Lipid | Fatty Acid Metabolism (Acyl Carnitine, Long Chain Saturated) | HMDB0000848 | HMDB0000848 |  |
| Cis-3,4-methyleneheptanoylcarnitine levels | -3.001 | 0.393 | 2.35E-14 | 0.651 | 0.099 | Lipid | Fatty Acid Metabolism (Acyl Carnitine, Medium Chain) |  | 0 |  |
| 5-dodecenoylcarnitine (C12:1) levels | 1.899 | 0.390 | 1.14E-06 | 0.862 | 0.423 | Lipid | Fatty Acid Metabolism (Acyl Carnitine, Monounsaturated) | HMDB13326 | HMDB13326 |  |
| Hexanoylglycine levels | 3.008 | 0.464 | 8.92E-11 | 0.941 | 0.158 | Lipid | Fatty Acid Metabolism (Acyl Glycine) | HMDB0000701 | HMDB0000701 |  |
| Picolinoylglycine levels | -1.961 | 0.394 | 6.45E-07 | 0.940 | 0.190 | Lipid | Fatty Acid Metabolism (Acyl Glycine) | HMDB0059766 | HMDB0059766 |  |
| Butyrylglycine levels | 2.590 | 0.467 | 2.84E-08 | 0.893 | 0.447 | Lipid | Fatty Acid Metabolism (also BCAA Metabolism) | HMDB0000808 | HMDB0000808 |  |
| 2-aminoheptanoate levels | -3.048 | 0.394 | 1.07E-14 | 0.542 | 0.204 | Lipid | Fatty Acid, Amino | HMDB0094649 | HMDB0094649 |  |
| (16 or 17)-methylstearate (a19:0 or i19:0) levels | 2.249 | 0.393 | 1.04E-08 | 0.649 | 0.293 | Lipid | Fatty Acid, Branched | HMDB0037397 | HMDB0037397 |  |
| Cis 3,4-methyleneheptanoate levels | -2.309 | 0.398 | 6.57E-09 | 0.683 | 0.119 | Lipid | Fatty Acid, Branched |  | 0 |  |
| Pristanate levels | 1.918 | 0.452 | 2.21E-05 | 0.882 | 0.401 | Lipid | Fatty Acid, Branched | HMDB0000795 | HMDB0000795 |  |
| Octadecanedioate levels | 2.031 | 0.481 | 2.45E-05 | 0.147 | 0.754 | Lipid | Fatty Acid, Dicarboxylate | HMDB0000782 | HMDB0000782 |  |
| 12,13-DiHOME levels | -1.747 | 0.402 | 1.41E-05 | 0.862 | 0.704 | Lipid | Fatty Acid, Dihydroxy | HMDB0004705 | HMDB0004705 | C14829 |
| Glycosyl-N-tricosanoyl-sphingadienine (d18:2/23:0) levels | 1.755 | 0.417 | 2.56E-05 | 0.995 | 0.665 | Lipid | Hexosylceramides (HCER) |  | 0 |  |
| Myristoleate (14:1n5) levels | 1.771 | 0.375 | 2.29E-06 | 0.402 | 0.582 | Lipid | Long Chain Monounsaturated Fatty Acid | HMDB0002000 | HMDB0002000 | C08322 |
| 10-nonadecenoate (19:1n9) levels | 1.781 | 0.387 | 4.11E-06 | 0.743 | 0.407 | Lipid | Long Chain Monounsaturated Fatty Acid | HMDB0013622 | HMDB0013622 |  |
| Erucate (22:1n9) levels | 2.078 | 0.398 | 1.73E-07 | 0.752 | 0.222 | Lipid | Long Chain Monounsaturated Fatty Acid | HMDB0002068 | HMDB0002068 | C08316 |
| Docosatrienoate (22:3n3) levels | 1.955 | 0.452 | 1.51E-05 | 0.795 | 0.347 | Lipid | Long Chain Polyunsaturated Fatty Acid (n3 and n6) | HMDB0002823 | HMDB0002823 | C16534 |
| Heneicosapentaenoate (21:5n3) levels | 2.992 | 0.519 | 7.93E-09 | 0.631 | 0.508 | Lipid | Long Chain Polyunsaturated Fatty Acid (n3 and n6) |  | 0 |  |
| Stearate (18:0) levels | 1.727 | 0.395 | 1.21E-05 | 0.948 | 0.513 | Lipid | Long Chain Saturated Fatty Acid | HMDB0000827 | HMDB0000827 | C01530 |
| Nonadecanoate (19:0) levels | 1.890 | 0.397 | 1.93E-06 | 0.894 | 0.268 | Lipid | Long Chain Saturated Fatty Acid | HMDB0000772 | HMDB0000772 | C16535 |
| Pentadecanoate (15:0) levels | 1.789 | 0.385 | 3.30E-06 | 0.764 | 0.211 | Lipid | Long Chain Saturated Fatty Acid | HMDB0000826 | HMDB0000826 | C16537 |
| Margarate (17:0) levels | 1.787 | 0.391 | 4.83E-06 | 0.855 | 0.396 | Lipid | Long Chain Saturated Fatty Acid | HMDB0002259 | HMDB0002259 |  |
| 1-linoleoyl-GPG (18:2) levels | -1.778 | 0.402 | 9.82E-06 | 0.916 | 0.399 | Lipid | Lysophospholipid |  | 0 |  |
| 5-dodecenoate (12:1n7) levels | 1.595 | 0.370 | 1.58E-05 | 0.531 | 0.651 | Lipid | Medium Chain Fatty Acid | HMDB0000529 | HMDB0000529 |  |
| Caprate (10:0) levels | 1.624 | 0.391 | 3.29E-05 | 0.960 | 0.701 | Lipid | Medium Chain Fatty Acid | HMDB0000511 | HMDB0000511 | C01571 |
| Caproate (6:0) levels | 2.933 | 0.471 | 4.87E-10 | 0.237 | 0.236 | Lipid | Medium Chain Fatty Acid | HMDB0000535 | HMDB0000535 | C01585 |
| 2-linoleoylglycerol (18:2) levels | -2.140 | 0.456 | 2.66E-06 | 0.951 | 0.602 | Lipid | Monoacylglycerol | HMDB0011538 | HMDB0011538 |  |
| 1-stearoyl-2-oleoyl-GPE (18:0/18:1) levels | -1.701 | 0.398 | 1.95E-05 | 0.811 | 0.251 | Lipid | Phosphatidylethanolamine (PE) | HMDB0008993 | HMDB0008993 |  |
| Choline levels | -1.676 | 0.382 | 1.12E-05 | 0.967 | 0.742 | Lipid | Phospholipid Metabolism | HMDB0000097 | HMDB0000097 | C00114 |
| 1-(1-enyl-palmitoyl)-2-arachidonoyl-GPE (p-16:0/20:4) levels | -1.683 | 0.397 | 2.23E-05 | 0.867 | 0.299 | Lipid | Plasmalogen | HMDB0011352 | HMDB0011352 |  |
| 1-(1-enyl-palmitoyl)-2-palmitoleoyl-GPC (P-16:0/16:1) levels | 1.741 | 0.387 | 6.78E-06 | 0.917 | 0.507 | Lipid | Plasmalogen | HMDB0011207 | HMDB0011207 |  |
| 3b-hydroxy-5-cholenoic acid levels | 1.984 | 0.439 | 6.10E-06 | 0.656 | 0.909 | Lipid | Secondary Bile Acid Metabolism | HMDB0000308 | HMDB0000308 |  |
| Sphingomyelin (d18:1/24:1, d18:2/24:0) levels | 2.370 | 0.390 | 1.16E-09 | 0.956 | 0.649 | Lipid | Sphingomyelins | HMDB0012107 | HMDB0012107 |  |
| Sphingomyelin (d18:1/19:0, d19:1/18:0) levels | -1.588 | 0.373 | 2.03E-05 | 0.673 | 0.907 | Lipid | Sphingomyelins |  | 0 |  |
| Sphingosine levels | -1.815 | 0.407 | 8.09E-06 | 0.876 | 0.670 | Lipid | Sphingosines | HMDB0000252 | HMDB0000252 | C00319 |
| 3beta-hydroxy-5-cholestenoate levels | 2.318 | 0.401 | 7.57E-09 | 0.732 | 0.163 | Lipid | Sterol |  | HMDB0012453 | C17333 |
| Beta-alanine levels | -2.692 | 0.403 | 2.27E-11 | 0.817 | 0.091 | Nucleotide | Pyrimidine Metabolism, Uracil containing | HMDB0000056 | HMDB0000056 | C00099 |
| Glutamine degradant levels | 1.913 | 0.333 | 9.19E-09 | 0.650 | 0.121 | Partially Characterized Molecules | Partially Characterized Molecules |  | 0 |  |
| Glycine conjugate of C10H14O2 (1) levels | -1.714 | 0.402 | 2.00E-05 | 0.713 | 0.395 | Partially Characterized Molecules | Partially Characterized Molecules |  | 0 |  |
| Branched-chain, straight-chain, or cyclopropyl 10:1 fatty acid (1) levels | 2.250 | 0.387 | 5.99E-09 | 0.889 | 0.551 | Partially Characterized Molecules | Partially Characterized Molecules |  | 0 |  |
| Bilirubin degradation product, C17H18N2O4 (2) levels | 1.645 | 0.386 | 1.99E-05 | 0.763 | 0.539 | Partially Characterized Molecules | Partially Characterized Molecules |  |  |  |
| 4-hydroxyphenylacetylglutamine levels | -1.865 | 0.390 | 1.72E-06 | 0.891 | 0.201 | Peptide | Acetylated Peptides |  | HMDB0006061 |  |
| Gamma-glutamylisoleucine levels | -2.214 | 0.384 | 8.08E-09 | 0.720 | 0.173 | Peptide | Gamma-glutamyl Amino Acid | HMDB0011170 | HMDB0011170 |  |
| 4-hydroxyhippurate levels | -3.245 | 0.388 | 5.77E-17 | 0.487 | 0.625 | Xenobiotics | Benzoate Metabolism | HMDB0013678 | HMDB0013678 |  |
| O-cresol sulfate levels | 2.044 | 0.424 | 1.46E-06 | 0.952 | 0.349 | Xenobiotics | Benzoate Metabolism | HMDB0011635 | HMDB0011635 |  |
| 2-hydroxyhippurate levels | -1.793 | 0.402 | 8.03E-06 | 0.841 | 0.628 | Xenobiotics | Benzoate Metabolism | HMDB0000840 | HMDB0000840 | C07588 |
| 3-methyl catechol sulfate (1) levels | 1.735 | 0.398 | 1.34E-05 | 0.867 | 0.494 | Xenobiotics | Benzoate Metabolism |  | 0 |  |
| 3-(3-hydroxyphenyl)propionate sulfate levels | -2.198 | 0.483 | 5.41E-06 | 0.699 | 0.477 | Xenobiotics | Benzoate Metabolism | HMDB0094710 | HMDB0094710 |  |
| 2-methoxyhydroquinone sulfate (1) levels | -1.968 | 0.413 | 1.91E-06 | 0.826 | 0.272 | Xenobiotics | Benzoate Metabolism |  |  |  |
| 6-hydroxyindole sulfate levels | -2.367 | 0.399 | 3.02E-09 | 0.418 | 0.377 | Xenobiotics | Chemical | HMDB0000682 | HMDB0000682 |  |
| Dibutyl sulfosuccinate levels | 1.780 | 0.406 | 1.17E-05 | 0.937 | 0.194 | Xenobiotics | Chemical |  | 0 |  |
| Homostachydrine levels | 1.659 | 0.389 | 1.97E-05 | 0.998 | 0.491 | Xenobiotics | Food Component/Plant | HMDB0033433 | HMDB0033433 | C08283 |
| (2,4 or 2,5)-dimethylphenol sulfate levels | 2.487 | 0.504 | 7.93E-07 | 0.832 | 0.623 | Xenobiotics | Food Component/Plant |  | 0 |  |
| 5-hydroxymethyl-2-furoylcarnitine levels | -2.284 | 0.510 | 7.61E-06 | 0.995 | 0.449 | Xenobiotics | Food Component/Plant |  | 0 |  |
| Theobromine levels | 2.129 | 0.403 | 1.26E-07 | 0.990 | 0.926 | Xenobiotics | Xanthine Metabolism | HMDB0002825 | HMDB0002825 | C07480 |
| 3-methylxanthine levels | 1.911 | 0.403 | 2.08E-06 | 0.986 | 0.790 | Xenobiotics | Xanthine Metabolism | HMDB0001886 | HMDB0001886 | C16357 |
| 7-methylxanthine levels | 2.102 | 0.407 | 2.44E-07 | 0.986 | 0.644 | Xenobiotics | Xanthine Metabolism | HMDB0001991 | HMDB0001991 | C16353 |
| X-12221 levels | -2.075 | 0.455 | 5.14E-06 | 0.370 | 0.539 |  |  |  |  |  |
| X-12117 levels | -1.913 | 0.389 | 8.95E-07 | 0.793 | 0.241 |  |  |  |  |  |
| X-12101 levels | -1.847 | 0.389 | 2.01E-06 | 0.896 | 0.163 |  |  |  |  |  |
| X-12411 levels | -2.166 | 0.382 | 1.45E-08 | 0.459 | 0.068 |  |  |  |  |  |
| X-12812 levels | -2.632 | 0.451 | 5.19E-09 | 0.758 | 0.613 |  |  |  |  |  |
| X-13507 levels | -2.417 | 0.402 | 1.83E-09 | 0.664 | 0.392 |  |  |  |  |  |
| X-13729 levels | -1.684 | 0.401 | 2.65E-05 | 0.471 | 0.450 |  |  |  |  |  |
| X-13728 levels | 2.213 | 0.410 | 6.54E-08 | 0.975 | 0.485 |  |  |  |  |  |
| X-13695 levels | -2.443 | 0.444 | 3.86E-08 | 0.584 | 0.060 |  |  |  |  |  |
| X-17146 levels | -1.970 | 0.415 | 2.05E-06 | 0.967 | 0.418 |  |  |  |  |  |
| X-17010 levels | -2.402 | 0.410 | 4.84E-09 | 0.983 | 0.760 |  |  |  |  |  |
| X-15503 levels | -1.689 | 0.363 | 3.27E-06 | 0.589 | 0.387 |  |  |  |  |  |
| X-18888 levels | -1.992 | 0.419 | 1.97E-06 | 0.999 | 0.752 |  |  |  |  |  |
| X-21319 levels | -2.044 | 0.403 | 3.80E-07 | 0.831 | 0.169 |  |  |  |  |  |
| X-21258 levels | 2.246 | 0.403 | 2.60E-08 | 0.856 | 0.358 |  |  |  |  |  |
| X-18913 levels | -1.783 | 0.398 | 7.52E-06 | 0.691 | 0.661 |  |  |  |  |  |
| X-21310 levels | -1.929 | 0.411 | 2.73E-06 | 0.459 | 0.391 |  |  |  |  |  |
| X-21442 levels | 2.941 | 0.435 | 1.31E-11 | 0.996 | 0.915 |  |  |  |  |  |
| X-23639 levels | 1.889 | 0.402 | 2.55E-06 | 0.922 | 0.697 |  |  |  |  |  |
| X-23587 levels | -3.355 | 0.415 | 5.86E-16 | 0.972 | 0.207 |  |  |  |  |  |
| X-23593 levels | -2.332 | 0.386 | 1.48E-09 | 0.943 | 0.286 |  |  |  |  |  |
| X-23680 levels | -1.728 | 0.392 | 1.03E-05 | 0.692 | 0.451 |  |  |  |  |  |
| X-23974 levels | 2.229 | 0.482 | 3.77E-06 | 0.192 | 0.943 |  |  |  |  |  |
| X-23782 levels | 1.666 | 0.386 | 1.58E-05 | 0.748 | 0.816 |  |  |  |  |  |
| X-24518 levels | -2.370 | 0.443 | 8.78E-08 | 0.876 | 0.279 |  |  |  |  |  |
| X-24585 levels | -1.633 | 0.389 | 2.76E-05 | 0.884 | 0.124 |  |  |  |  |  |
| X-25371 levels | -2.645 | 0.395 | 2.21E-11 | 0.546 | 0.131 |  |  |  |  |  |
| X-25433 levels | -1.987 | 0.451 | 1.07E-05 | 0.917 | 0.235 |  |  |  |  |  |
| X-12112 levels | -1.843 | 0.402 | 4.47E-06 | 0.986 | 0.270 |  |  |  |  |  |
| 3-phosphoglycerate to phosphate ratio | -1.799 | 0.397 | 5.99E-06 | 0.999 | 0.857 |  |  |  |  |  |
| Adenosine 5'-monophosphate (AMP) to palmitate (16:0) ratio | -2.585 | 0.397 | 7.22E-11 | 0.603 | 0.912 |  |  |  |  |  |
| Adenosine 5'-monophosphate (AMP) to phosphate ratio | -1.726 | 0.397 | 1.37E-05 | 0.661 | 0.357 |  |  |  |  |  |
| Sphingosine to phosphate ratio | -1.852 | 0.405 | 4.75E-06 | 0.877 | 0.738 |  |  |  |  |  |
| Inosine 5'-monophosphate (IMP) to phosphate ratio | -2.986 | 0.579 | 2.51E-07 | 0.541 | 0.793 |  |  |  |  |  |
| Glucose to maltose ratio | 2.696 | 0.453 | 2.63E-09 | 0.844 | 0.891 |  |  |  |  |  |
| Creatine to carnitine ratio | -1.763 | 0.362 | 1.12E-06 | 0.885 | 0.581 |  |  |  |  |  |
| Spermidine to (N(1) + N(8))-acetylspermidine ratio | -1.805 | 0.421 | 1.82E-05 | 0.977 | 0.218 |  |  |  |  |  |
| 3-phosphoglycerate to glycerate ratio | -1.685 | 0.401 | 2.64E-05 | 0.992 | 0.848 |  |  |  |  |  |
| Adenosine 5'-monophosphate (AMP) to tryptophan ratio | -1.737 | 0.393 | 9.78E-06 | 0.649 | 0.422 |  |  |  |  |  |
| Adenosine 5'-monophosphate (AMP) to arginine ratio | -1.745 | 0.396 | 1.03E-05 | 0.765 | 0.374 |  |  |  |  |  |
| Adenosine 5'-monophosphate (AMP) to asparagine ratio | -1.723 | 0.396 | 1.38E-05 | 0.849 | 0.390 |  |  |  |  |  |
| Isoleucine to phosphate ratio | -2.348 | 0.375 | 3.98E-10 | 0.910 | 0.784 |  |  |  |  |  |
| Cysteinylglycine to taurine ratio | 2.094 | 0.388 | 6.60E-08 | 0.916 | 0.172 |  |  |  |  |  |
| Phenylpyruvate to 4-hydroxyphenylpyruvate ratio | 1.864 | 0.402 | 3.44E-06 | 0.819 | 0.551 |  |  |  |  |  |
| Cholesterol to cortisol ratio | -1.973 | 0.387 | 3.55E-07 | 0.963 | 0.400 |  |  |  |  |  |
| Citrate to 4-hydroxyphenylpyruvate ratio | 2.475 | 0.410 | 1.50E-09 | 0.987 | 0.446 |  |  |  |  |  |
| Salicylate to caprylate (8:0) ratio | -2.032 | 0.399 | 3.61E-07 | 0.988 | 0.440 |  |  |  |  |  |
| Inosine to EDTA ratio | -2.219 | 0.515 | 1.63E-05 | 0.699 | 0.729 |  |  |  |  |  |
| Phosphate to oleoyl-linoleoyl-glycerol (18:1 to 18:2) [2] ratio | 2.733 | 0.401 | 9.73E-12 | 0.792 | 0.974 |  |  |  |  |  |
| Phosphate to linoleoyl-arachidonoyl-glycerol (18:2 to 20:4) [1] ratio | 2.166 | 0.414 | 1.64E-07 | 1.000 | 0.603 |  |  |  |  |  |
| Retinol (Vitamin A) to linoleoyl-arachidonoyl-glycerol (18:2 to 20:4) [1] ratio | 2.357 | 0.415 | 1.38E-08 | 0.999 | 0.516 |  |  |  |  |  |
| Retinol (Vitamin A) to oleoyl-linoleoyl-glycerol (18:1 to 18:2) [2] ratio | 2.108 | 0.404 | 1.87E-07 | 0.903 | 0.826 |  |  |  |  |  |
| Glycerol to mannitol to sorbitol ratio | 2.325 | 0.390 | 2.61E-09 | 0.970 | 0.437 |  |  |  |  |  |
| Carnitine to propionylcarnitine (C3) ratio | 1.950 | 0.389 | 5.26E-07 | 0.969 | 0.684 |  |  |  |  |  |
| Adenosine 5'-diphosphate (ADP) to mannitol to sorbitol ratio | 2.996 | 0.540 | 2.82E-08 | 0.922 | 0.181 |  |  |  |  |  |
| Glucose to mannitol to sorbitol ratio | 1.761 | 0.395 | 8.18E-06 | 0.936 | 0.611 |  |  |  |  |  |
| Inosine 5'-monophosphate (IMP) to urate ratio | -2.428 | 0.563 | 1.59E-05 | 0.893 | 0.849 |  |  |  |  |  |
| Leucine to phosphate ratio | -1.775 | 0.369 | 1.49E-06 | 0.999 | 0.774 |  |  |  |  |  |
| Cholesterol to linoleoyl-arachidonoyl-glycerol (18:2 to 20:4) [1] ratio | 2.368 | 0.411 | 8.12E-09 | 0.995 | 0.433 |  |  |  |  |  |
| Cholesterol to oleoyl-linoleoyl-glycerol (18:1 to 18:2) [2] ratio | -2.785 | 0.398 | 2.68E-12 | 0.804 | 0.710 |  |  |  |  |  |
| Benzoate to oleoyl-linoleoyl-glycerol (18:1 to 18:2) [2] ratio | 1.880 | 0.427 | 1.05E-05 | 0.998 | 0.547 |  |  |  |  |  |
| Benzoate to linoleoyl-arachidonoyl-glycerol (18:2 to 20:4) [2] ratio | 1.889 | 0.436 | 1.48E-05 | 0.924 | 0.277 |  |  |  |  |  |
| Salicylate to citrate ratio | -1.712 | 0.400 | 1.85E-05 | 0.987 | 0.455 |  |  |  |  |  |
| Maltose to sucrose ratio | -2.316 | 0.472 | 9.05E-07 | 0.971 | 0.703 |  |  |  |  |  |
| Fructose to maltose ratio | 2.453 | 0.456 | 7.42E-08 | 0.984 | 0.250 |  |  |  |  |  |
| 3-methyl-2-oxovalerate to 4-methyl-2-oxopentanoate ratio | -2.379 | 0.398 | 2.28E-09 | 0.780 | 0.436 |  |  |  |  |  |
| 3-methyl-2-oxovalerate to 3-methyl-2-oxobutyrate ratio | -2.557 | 0.393 | 7.33E-11 | 0.816 | 0.732 |  |  |  |  |  |

Supplementary Table 6. The effects of SGLT2 inhibition on circulating metabolites and the effects of metabolites on CSVD manifestations.

*beta1, se1, pvalue1, Q_pval1, pleiotropy1 reflect the effects of SGLT2 inhibition on circulating metabolites (mediator), while beta2, se2, pvalue2, Q_pval2, pleiotropy2 reflect the effects of metabolites on CSVD manifestations (Outcome).

| Exposure | Mediator | Outcome | beta1 | se1 | pvalue1 | Q_pval1 | pleiotropy1 | beta2 | se2 | pvalue2 | Q_pval2 | pleiotropy2 |
| --- | --- | --- | --- | --- | --- | --- | --- | --- | --- | --- | --- | --- |
| SGLT2 inhibition | Phenol sulfate levels | SVS | -2.063 | 0.403 | 3.12E-07 | 0.858 | 0.551 | -0.187 | 0.076 | 1.34E-02 | 0.684 | 0.697 |
| SGLT2 inhibition | Dibutyl sulfosuccinate levels | SVS | 1.780 | 0.406 | 1.17E-05 | 0.937 | 0.194 | -0.171 | 0.078 | 2.80E-02 | 0.106 | 0.487 |
| SGLT2 inhibition | 4-acetamidobutanoate levels | SVS | 2.216 | 0.379 | 4.93E-09 | 0.966 | 0.357 | -0.250 | 0.057 | **9.76E-06** | 0.886 | 0.840 |
| SGLT2 inhibition | Creatine levels | SVS | -1.634 | 0.365 | 7.57E-06 | 0.950 | 0.320 | -0.223 | 0.085 | 8.73E-03 | 0.237 | 0.218 |
| SGLT2 inhibition | Sphingosine levels | SVS | -1.815 | 0.407 | 8.09E-06 | 0.876 | 0.670 | -0.252 | 0.081 | 1.87E-03 | 0.247 | 0.369 |
| SGLT2 inhibition | Adenosine 5'-monophosphate (AMP) to arginine ratio | SVS | -1.745 | 0.396 | 1.03E-05 | 0.765 | 0.374 | -0.195 | 0.099 | 4.87E-02 | 0.893 | 0.308 |
| SGLT2 inhibition | Phenylpyruvate to 4-hydroxyphenylpyruvate ratio | SVS | 1.864 | 0.402 | 3.44E-06 | 0.819 | 0.551 | -0.224 | 0.070 | 1.41E-03 | 0.879 | 0.944 |
| SGLT2 inhibition | 7-methylxanthine levels | PWMH | 2.102 | 0.407 | 2.44E-07 | 0.986 | 0.644 | 0.133 | 0.038 | 4.51E-04 | 0.748 | 0.747 |
| SGLT2 inhibition | 3-methylxanthine levels | PWMH | 1.911 | 0.403 | 2.08E-06 | 0.986 | 0.790 | 0.064 | 0.032 | 4.59E-02 | 0.199 | 0.363 |
| SGLT2 inhibition | X-23680 levels | DWMH | -1.728 | 0.392 | 1.03E-05 | 0.692 | 0.451 | -0.106 | 0.032 | 8.62E-04 | 0.580 | 0.245 |
| SGLT2 inhibition | 1-linoleoyl-GPG (18:2) levels | DWMH | -1.778 | 0.402 | 9.82E-06 | 0.916 | 0.399 | -0.059 | 0.028 | 3.89E-02 | 0.883 | 0.711 |
| SGLT2 inhibition | Phenylpyruvate to 4-hydroxyphenylpyruvate ratio | DWMH | 1.864 | 0.402 | 3.44E-06 | 0.819 | 0.551 | -0.079 | 0.040 | 4.93E-02 | 0.115 | 0.597 |
| SGLT2 inhibition | X-23639 levels | DWMH | 1.889 | 0.402 | 2.55E-06 | 0.922 | 0.697 | 0.080 | 0.038 | 3.69E-02 | 0.814 | 0.149 |
| SGLT2 inhibition | 7-methylxanthine levels | DWMH | 2.102 | 0.407 | 2.44E-07 | 0.986 | 0.644 | 0.131 | 0.047 | 5.82E-03 | 0.119 | 0.568 |
| SGLT2 inhibition | Sphingomyelin (d18:1/24:1, d18:2/24:0) levels | DWMH | 2.370 | 0.390 | 1.16E-09 | 0.956 | 0.649 | 0.106 | 0.053 | 4.35E-02 | 0.254 | 0.343 |
| SGLT2 inhibition | Cis-3,4-methyleneheptanoylcarnitine levels | WM_MD | -3.001 | 0.393 | 2.35E-14 | 0.651 | 0.099 | -0.037 | 0.017 | 3.20E-02 | 0.450 | 0.200 |
| SGLT2 inhibition | Cholesterol to oleoyl-linoleoyl-glycerol (18:1 to 18:2) [2] ratio | WM_MD | 2.785 | 0.398 | 2.68E-12 | 0.804 | 0.710 | -0.065 | 0.021 | 1.89E-03 | 0.051 | 0.902 |
| SGLT2 inhibition | 4-acetamidobutanoate levels | WM_MD | 2.216 | 0.379 | 4.93E-09 | 0.966 | 0.357 | 0.048 | 0.019 | 1.27E-02 | 0.313 | 0.485 |
| SGLT2 inhibition | Cysteine s-sulfate levels | WM_MD | 2.167 | 0.392 | 3.34E-08 | 0.569 | 0.141 | 0.060 | 0.025 | 1.63E-02 | 0.629 | 0.697 |
| SGLT2 inhibition | Myristoleate (14:1n5) levels | WM_MD | 1.771 | 0.375 | 2.29E-06 | 0.402 | 0.582 | -0.070 | 0.025 | 4.47E-03 | 0.390 | 0.303 |
| SGLT2 inhibition | X-23782 levels | WM_MD | 1.666 | 0.386 | 1.58E-05 | 0.748 | 0.816 | -0.062 | 0.028 | 2.81E-02 | 0.134 | 0.134 |
| SGLT2 inhibition | 1-(1-enyl-palmitoyl)-2-arachidonoyl-GPE (p-16:0/20:4) levels | WM_MD | -1.683 | 0.397 | 2.23E-05 | 0.867 | 0.299 | -0.084 | 0.032 | 8.11E-03 | 0.062 | 0.654 |
| SGLT2 inhibition | Octadecanedioate levels | WM_MD | 2.031 | 0.481 | 2.45E-05 | 0.147 | 0.754 | -0.040 | 0.018 | 2.88E-02 | 0.403 | 0.241 |
| SGLT2 inhibition | X-23587 levels | WM_AD | -3.355 | 0.415 | 5.86E-16 | 0.972 | 0.207 | -0.045 | 0.023 | 4.53E-02 | 0.665 | 0.798 |
| SGLT2 inhibition | Stearoylcarnitine levels | WM_AD | 3.062 | 0.396 | 9.81E-15 | 0.921 | 0.299 | 0.051 | 0.020 | 9.50E-03 | 0.347 | 0.300 |
| SGLT2 inhibition | 4-acetamidobutanoate levels | WM_AD | 2.216 | 0.379 | 4.93E-09 | 0.966 | 0.357 | 0.067 | 0.022 | 1.76E-03 | 0.151 | 0.606 |
| SGLT2 inhibition | Cysteine s-sulfate levels | WM_AD | 2.167 | 0.392 | 3.34E-08 | 0.569 | 0.141 | 0.060 | 0.026 | 1.97E-02 | 0.431 | 0.739 |
| SGLT2 inhibition | N-acetylcitrulline levels | WM_AD | -2.194 | 0.421 | 1.89E-07 | 0.682 | 0.187 | -0.025 | 0.012 | 3.41E-02 | 0.261 | 0.253 |
| SGLT2 inhibition | 1-(1-enyl-palmitoyl)-2-arachidonoyl-GPE (p-16:0/20:4) levels | WM_AD | -1.683 | 0.397 | 2.23E-05 | 0.867 | 0.299 | -0.079 | 0.033 | 1.54E-02 | 0.072 | 0.883 |
| SGLT2 inhibition | Cis-3,4-methyleneheptanoylcarnitine levels | WM_RD | -3.001 | 0.393 | 2.35E-14 | 0.651 | 0.099 | -0.035 | 0.017 | 3.94E-02 | 0.451 | 0.341 |
| SGLT2 inhibition | Cholesterol to oleoyl-linoleoyl-glycerol (18:1 to 18:2) [2] ratio | WM_RD | -2.785 | 0.398 | 2.68E-12 | 0.804 | 0.710 | 0.075 | 0.020 | **1.91E-04** | 0.072 | 0.867 |
| SGLT2 inhibition | Cysteine s-sulfate levels | WM_RD | 2.167 | 0.392 | 3.34E-08 | 0.569 | 0.141 | 0.056 | 0.025 | 2.45E-02 | 0.657 | 0.595 |
| SGLT2 inhibition | Myristoleate (14:1n5) levels | WM_RD | 1.771 | 0.375 | 2.29E-06 | 0.402 | 0.582 | -0.079 | 0.024 | 1.16E-03 | 0.405 | 0.203 |
| SGLT2 inhibition | X-23782 levels | WM_RD | 1.666 | 0.386 | 1.58E-05 | 0.748 | 0.816 | -0.062 | 0.028 | 2.69E-02 | 0.139 | 0.107 |
| SGLT2 inhibition | 1-(1-enyl-palmitoyl)-2-arachidonoyl-GPE (p-16:0/20:4) levels | WM_RD | -1.683 | 0.397 | 2.23E-05 | 0.867 | 0.299 | -0.082 | 0.031 | 8.85E-03 | 0.063 | 0.555 |
| SGLT2 inhibition | Octadecanedioate levels | WM_RD | 2.031 | 0.481 | 2.45E-05 | 0.147 | 0.754 | -0.045 | 0.018 | 1.16E-02 | 0.668 | 0.516 |
| SGLT2 inhibition | 1-methyl-4-imidazoleacetate levels | deep_CMBs | -2.593 | 0.428 | 1.39E-09 | 0.254 | 0.097 | 0.300 | 0.135 | 2.61E-02 | 0.316 | 0.105 |
| SGLT2 inhibition | 2-linoleoylglycerol (18:2) levels | deep_CMBs | -2.140 | 0.456 | 2.66E-06 | 0.951 | 0.602 | -0.588 | 0.209 | 4.92E-03 | 0.914 | 0.661 |
| SGLT2 inhibition | 3beta-hydroxy-5-cholestenoate levels | deep_CMBs | 2.318 | 0.401 | 7.57E-09 | 0.732 | 0.163 | -0.331 | 0.140 | 1.83E-02 | 0.996 | 0.496 |
| SGLT2 inhibition | O-cresol sulfate levels | deep_CMBs | 2.044 | 0.424 | 1.46E-06 | 0.952 | 0.349 | -0.478 | 0.221 | 3.05E-02 | 0.913 | 0.408 |
| SGLT2 inhibition | X-13507 levels | deep_CMBs | -2.417 | 0.402 | 1.83E-09 | 0.664 | 0.392 | 0.516 | 0.255 | 4.26E-02 | 0.805 | 0.986 |
| SGLT2 inhibition | Cysteinylglycine to taurine ratio | deep_CMBs | 2.094 | 0.388 | 6.60E-08 | 0.916 | 0.172 | -0.499 | 0.182 | 6.04E-03 | 0.795 | 0.604 |
| SGLT2 inhibition | Leucine to phosphate ratio | deep_CMBs | -1.775 | 0.369 | 1.49E-06 | 0.999 | 0.774 | -0.522 | 0.233 | 2.52E-02 | 0.367 | 0.449 |
| SGLT2 inhibition | Adenosine 5'-monophosphate (AMP) to palmitate (16:0) ratio | WM_EPVS | -2.585 | 0.397 | 7.22E-11 | 0.603 | 0.912 | -0.023 | 0.012 | 4.74E-02 | 0.836 | 0.441 |
| SGLT2 inhibition | 3beta-hydroxy-5-cholestenoate levels | WM_EPVS | 2.318 | 0.401 | 7.57E-09 | 0.732 | 0.163 | -0.018 | 0.007 | 1.65E-02 | 0.747 | 0.293 |
| SGLT2 inhibition | 1-(1-enyl-palmitoyl)-2-palmitoleoyl-GPC (P-16:0/16:1) levels | WM_EPVS | 1.741 | 0.387 | 6.78E-06 | 0.917 | 0.507 | -0.015 | 0.007 | 2.67E-02 | 0.309 | 0.002 |
| SGLT2 inhibition | Creatine levels | WM_EPVS | -1.634 | 0.365 | 7.57E-06 | 0.950 | 0.320 | -0.024 | 0.010 | 2.10E-02 | 0.360 | 0.142 |
| SGLT2 inhibition | 1-linoleoyl-GPG (18:2) levels | WM_EPVS | -1.778 | 0.402 | 9.82E-06 | 0.916 | 0.399 | -0.023 | 0.008 | 3.39E-03 | 0.495 | 0.196 |
| SGLT2 inhibition | Benzoate to oleoyl-linoleoyl-glycerol (18:1 to 18:2) [2] ratio | WM_EPVS | 1.880 | 0.427 | 1.05E-05 | 0.998 | 0.547 | -0.016 | 0.008 | 4.37E-02 | 0.451 | 0.880 |
| SGLT2 inhibition | Glutamine degradant levels | BG_EPVS | 1.913 | 0.333 | 9.19E-09 | 0.650 | 0.121 | 0.025 | 0.010 | 1.34E-02 | 0.206 | 0.414 |
| SGLT2 inhibition | Indoleacetylglutamine levels | BG_EPVS | -2.389 | 0.449 | 1.02E-07 | 0.575 | 0.101 | -0.019 | 0.009 | 4.54E-02 | 0.055 | 0.330 |
| SGLT2 inhibition | Inosine 5'-monophosphate (IMP) to phosphate ratio | BG_EPVS | -2.986 | 0.579 | 2.51E-07 | 0.541 | 0.793 | 0.021 | 0.008 | 9.06E-03 | 0.681 | 0.388 |
| SGLT2 inhibition | N-lactoyl isoleucine levels | BG_EPVS | 1.981 | 0.405 | 1.03E-06 | 0.997 | 0.646 | 0.024 | 0.012 | 4.96E-02 | 0.520 | 0.853 |
| SGLT2 inhibition | S-methylcysteine levels | BG_EPVS | 1.839 | 0.398 | 3.74E-06 | 0.831 | 0.152 | -0.020 | 0.009 | 2.86E-02 | 0.996 | 0.945 |
| SGLT2 inhibition | X-12112 levels | BG_EPVS | -1.843 | 0.402 | 4.47E-06 | 0.986 | 0.270 | -0.010 | 0.005 | 3.46E-02 | 0.804 | 0.654 |
| SGLT2 inhibition | Choline levels | BG_EPVS | -1.676 | 0.382 | 1.12E-05 | 0.967 | 0.742 | 0.020 | 0.010 | 4.48E-02 | 0.615 | 0.395 |
| SGLT2 inhibition | Sphingomyelin (d18:1/24:1, d18:2/24:0) levels | HIP_EPVS | 2.370 | 0.390 | 1.16E-09 | 0.956 | 0.649 | -0.025 | 0.012 | 3.67E-02 | 0.678 | 0.164 |
| SGLT2 inhibition | 6-hydroxyindole sulfate levels | HIP_EPVS | -2.367 | 0.399 | 3.02E-09 | 0.418 | 0.377 | 0.024 | 0.012 | 4.98E-02 | 0.434 | 0.482 |
| SGLT2 inhibition | Cis 3,4-methyleneheptanoate levels | HIP_EPVS | -2.309 | 0.398 | 6.57E-09 | 0.683 | 0.119 | 0.024 | 0.010 | 1.77E-02 | 0.557 | 0.540 |
| SGLT2 inhibition | Hydantoin-5-propionate levels | HIP_EPVS | -2.278 | 0.398 | 1.05E-08 | 0.929 | 0.384 | -0.024 | 0.012 | 3.78E-02 | 0.786 | 0.434 |
| SGLT2 inhibition | Inosine 5'-monophosphate (IMP) to phosphate ratio | HIP_EPVS | -2.986 | 0.579 | 2.51E-07 | 0.541 | 0.793 | 0.023 | 0.009 | 1.07E-02 | 0.243 | 0.269 |
| SGLT2 inhibition | Oleoyl ethanolamide levels | HIP_EPVS | 1.988 | 0.393 | 4.24E-07 | 0.970 | 0.245 | -0.019 | 0.009 | 2.77E-02 | 0.983 | 0.532 |
| SGLT2 inhibition | Maltose to sucrose ratio | HIP_EPVS | -2.316 | 0.472 | 9.05E-07 | 0.971 | 0.703 | 0.024 | 0.010 | 2.13E-02 | 0.506 | 0.401 |
| SGLT2 inhibition | X-23639 levels | HIP_EPVS | 1.889 | 0.402 | 2.55E-06 | 0.922 | 0.697 | -0.034 | 0.010 | 6.72E-04 | 0.605 | 0.618 |
| SGLT2 inhibition | Adenosine 5'-monophosphate (AMP) to asparagine ratio | HIP_EPVS | -1.723 | 0.396 | 1.38E-05 | 0.849 | 0.390 | -0.024 | 0.011 | 2.69E-02 | 0.206 | 0.817 |
| SGLT2 inhibition | Homostachydrine levels | HIP_EPVS | 1.659 | 0.389 | 1.97E-05 | 0.998 | 0.491 | -0.031 | 0.009 | 6.50E-04 | 0.645 | 0.709 |
| SGLT2 inhibition | Lysine levels | HIP_EPVS | 1.674 | 0.399 | 2.70E-05 | 0.979 | 0.850 | 0.020 | 0.009 | 2.39E-02 | 0.184 | 0.275 |
